# High throughput techniques to reveal the molecular physiology and evolution of digestion in spiders

**DOI:** 10.1186/s12864-016-3048-9

**Published:** 2016-09-07

**Authors:** Felipe J. Fuzita, Martijn W. H. Pinkse, José S. L. Patane, Peter D. E. M. Verhaert, Adriana R. Lopes

**Affiliations:** 1 Laboratory of Biochemistry and Biophysics, Instituto Butantan, São Paulo, 05503-000 Brazil; 2 Biotechnology Program, University of São Paulo, São Paulo, Brazil; 3 Laboratory of Analytical Biotechnology and Innovative Peptide Biology, Delft University of Technology, Delft, The Netherlands; 4 Department of Biochemistry, Institute of Chemistry, University of São Paulo, São Paulo, Brazil; 5 Department of Biology, University of Antwerp, Antwerp, Belgium

**Keywords:** Astacin, Digestion, Enzyme, High throughput (-omics) techniques, Spider, Arachnida, *Nephilingis* (*Nephilengys cruentata*)

## Abstract

**Background:**

Spiders are known for their predatory efficiency and for their high capacity of digesting relatively large prey. They do this by combining both extracorporeal and intracellular digestion. Whereas many high throughput (“-omics”) techniques focus on biomolecules in spider venom, so far this approach has not yet been applied to investigate the protein composition of spider midgut diverticula (MD) and digestive fluid (DF).

**Results:**

We here report on our investigations of both MD and DF of the spider *Nephilingis* (*Nephilengys) cruentata* through the use of next generation sequencing and shotgun proteomics. This shows that the DF is composed of a variety of hydrolases including peptidases, carbohydrases, lipases and nuclease, as well as of toxins and regulatory proteins. We detect 25 astacins in the DF. Phylogenetic analysis of the corresponding transcript(s) in Arachnida suggests that astacins have acquired an unprecedented role for extracorporeal digestion in Araneae, with different orthologs used by each family. The results of a comparative study of spiders in distinct physiological conditions allow us to propose some digestion mechanisms in this interesting animal taxon.

**Conclusion:**

All the high throughput data allowed the demonstration that DF is a secretion originating from the MD. We identified enzymes involved in the extracellular and intracellular phases of digestion. Besides that, data analyses show a large gene duplication event in Araneae digestive process evolution, mainly of astacin genes. We were also able to identify proteins expressed and translated in the digestive system, which until now had been exclusively associated to venom glands.

**Electronic supplementary material:**

The online version of this article (doi:10.1186/s12864-016-3048-9) contains supplementary material, which is available to authorized users.

## Background

Spiders are efficient predators capable of capturing and ingesting relatively big preys [1, 2]. They manage this by virtue of a peculiar digestive system, which combines an extra-oral (EOD) process with intracellular digestion [3, 4]. In addition arachnids are able to survive long fasting periods, which is indicative of an efficient absorptive and reservoir process. Thus, spiders possess a very efficient digestive system, which is capable of hydrolyzing and storing as much food/nutrients as possible from one single meal. We find it remarkable that, with the current availability of powerful “-omics” techniques (next generation sequencing [NGS] and proteomics) allowing efficient high throughput analyses, so far there has not been any extensive study on the proteins/enzymes involved in spider digestion. It appears that the main focus on the study of spider biomolecules has been related to venom and silk glands [5].

Histologically, it has already been shown for a long time that shortly after prey capture secretory cells discharge their vesicles into the spider midgut lumen. After food uptake the digestive cells start the internalization of the predigested food by pinocytosis. The pinocytotic vesicles are then assembled, originating big digestive vacuoles in which intracellular digestion, both fast and slow, takes place [4]. Earlier biochemical characterization of spider digestive fluid identified the presence of peptidases [6–10], carbohydrases [11, 12], esterases, phosphatases and nucleases [13]. Despite the recent publication of two spider genomes [5], there is little or no information about digestive system-specific proteins and expression patterns correlated with distinct physiological conditions such as fasting and feeding periods.

The association of genome data with proteomic data yielded important insights about spider venom and silk gland composition in evolutionarily distinct taxa (Mygalomorphae and Araneomorphae). Furthermore, Sanggaard and co-workers [5] expressed the need for a digestive fluid investigation in order to better comprehend the digestive processes in spiders. Our work on the spider *Nephilingis* (*Nephilengys) cruentata* [14] focuses not only the digestive fluid (DF), but also on the opisthosomal midgut diverticula (MD). We investigated these in different feeding conditions by label-free quantitative shotgun proteomics. Using the Illumina® NGS platform a database was constructed to be used for protein identification, to verify differentially expressed genes in fasting and fed conditions, and to confirm that the DF most probably is a secretion originating from the MD. Astacins were identified and phylogenetically analyzed evincing a large gene duplication event in Araneae. We were also able to identify other proteins expressed and translated in the digestive system (e.g. venom peptide isomerase), which, until now, had been exclusively associated to venom glands. Finally, our approach allows us the proposal of a model for the digestive process in spiders, more complete than any published before, by showing the proteins involved in EOD and intracellular digestion as well as candidate molecules involved in the endocytic pathway.

## Results

### DF and MD transcriptome and proteome data

**Fig. 1:**
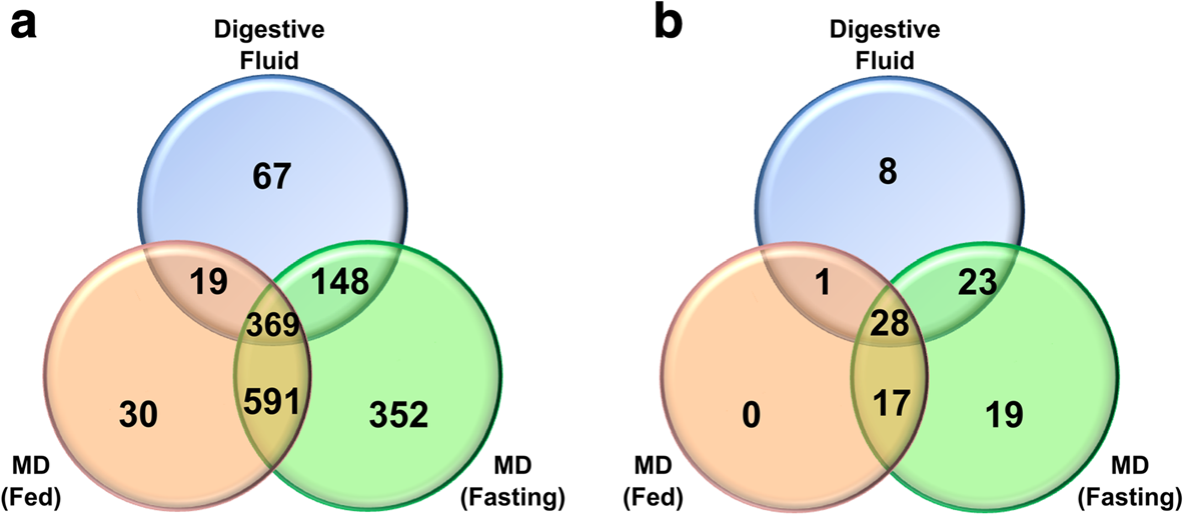
Venn diagram of proteome data of digestive fluid (DF) and opisthosomal midgut diverticula (MD) samples of *N. cruentata*. **a** All identified proteins in DF and MD from fasting and 9 h fed spiders. **b** Digestive enzymes identified in DF and MD samples. All DF samples obtained from fasting and different periods of feeding (1, 3, 9, 25 and 48 h) were used

RNA-seq analysis of MD samples from three distinct physiological conditions (fasting, 1 and 9 h fed) resulted in a total of 23,249 contigs after data assembly (Additional file 1). This transcriptome shotgun assembly project has been deposited at DDJB/EMBL/GenBank under the accession GEWZ01000000. Additional file 2 exhibits some parameters of the individual assemblies. From these contigs at least 60 % did not yield BLAST homology hits and were annotated as unknown proteins, as was already observed in other transcriptome studies [15]. The transcriptome final translated sequences were used to generate a database (Additional file 3), which supported a high quality identification of the peptides obtained at the proteomic analyses (at least two identified peptides were required for a positive identification, with a false discovery rate of 0.1 %; Fig. 1a and Additional file 4). The use of this transcriptome database allowed the quantification by shotgun proteomics of 393 proteins from the *N. cruentata* DF, 1359 proteins from the MD of fasting spiders and 779 proteins from the MD samples of fed animals. The qualitative global number of identification per physiological sample (DF, fasting and fed spiders) is shown in Fig. 1a. As explained in item 4.4 qualitative and quantitative number of identified proteins may vary. The sum of distinct proteins from MD of fed and fasting animals resulted in the identification of 1571 proteins. From these proteins, 12 % align with sequences annotated as unknown proteins, and this was also true for 20 % of the protein sequences from the DF. The identification of this large number of proteins in the DF using a database generated from the MD RNA-seq represents the first molecular evidence that the DF is originally synthesized in the MD. The DF proteins qualitatively correspond to 25 % of the proteins identified in the MD. This is a representative protein quantity as DF would be the product of MD secretion.

Gene ontology (GO) analyses of the DF proteome indicate that its composition reflects processes beyond digestion, enzymes and peptidase inhibitors. Typical membrane proteins involved in vesicle mediated transport; membrane transporters, lipid binding, ion binding, and stress response are also present in the DF (Additional file 5), which could be an evidence of the secretory process in spiders.

### Digestive enzymes identified by shotgun proteomics

A Venn diagram (Fig. 1a) shows the number of total proteins identified with the shotgun mass spectrometry (MS) strategy per sample analyzed from the spider *N. cruentata* (DF and MD from fasting and fed animals). The shared proteins are also evidenced and this result indicates that DF presents 89 % of shared proteins with the sum of MD samples. This fact corroborates the previous evidences that DF is a product of secretion of MD.

**Fig. 2:**
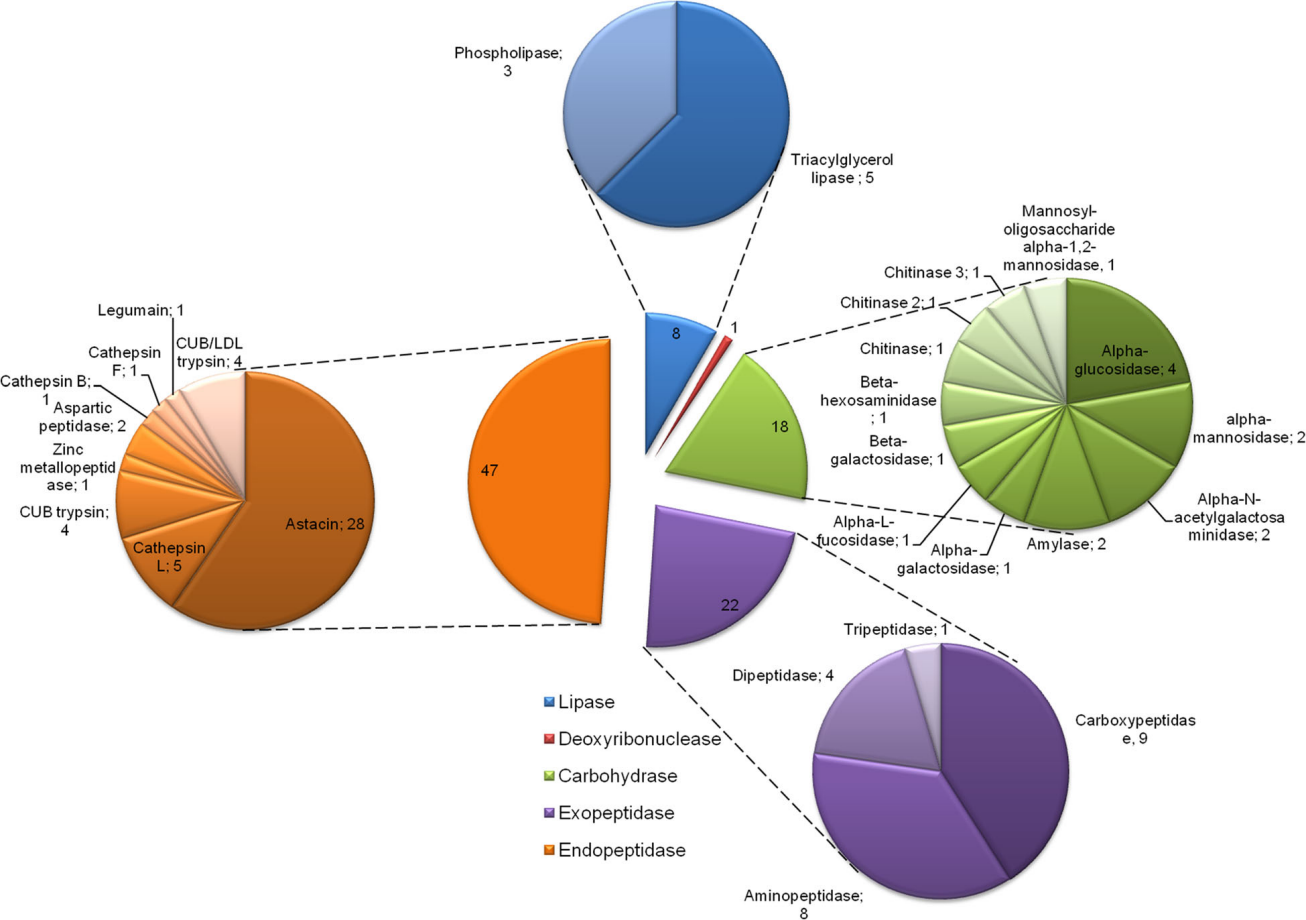
Sum of all distinct elected digestive enzyme types and respective number of isoforms identified by shotgun proteomics in MD from fed and fast animals and DF

A digestive role was attributed to a total of 96 proteins identified in the MD and the DF from the spider *N. cruentata* (Table 1). Although a precise identification of which enzymes are involved in food digestion is tricky, it is quite plausible to assume that secreted hydrolases and proteins associated with a lysosome-like organelle may be possible digestive enzymes. Table 1 shows the identified digestive enzymes. Twenty-eight proteins are present in the three samples (DF, fasting MD, fed MD). We identified 17 common sequences between fed and fasting animals, and 23 shared between the DF and the fasting condition. Some proteins were exclusively identified in an individual sample source: 19 were only found in fasting spiders and, finally, 8 hydrolases were exclusively detected in the DF (Table 1 and Fig. 1b). The numbers of different enzyme types from Table 1 are more clearly visualized in Fig. 2. Endopeptidases appear to be the most abundant enzymes, represented by 47 sequences. Exopeptidases, carbohydrases and lipases had 22, 18 and 8 different sequences respectively. One deoxyribonuclease was also identified (Fig. 2). The majority of the endopeptidases are astacin-like metallopeptidases (Table 1 and Additional file 6).

**Table 1:** Probable digestive enzymes identified by mass spectrometry in the samples of the digestive fluid, fasting and fed MD

3 conditions (28)	Fasting and Fed (17)	Fasting and Digestive Fluid (23)	Fasting (19)	Digestive Fluid (8)
Astacin 11	Astacin 26	Astacin 10	Cathepsin L 4	Astacin 25
Astacin 16	Astacin 36	Astacin 15	Astacin 24	Astacin 32
Astacin 18	Aspartic peptidase 3	Astacin 17	Dipeptidyl peptidase 2	Astacin 6
Astacin 19	Cathepsin L1	Astacin 23	Tripeptidyl peptidase 2	CUB/LDL trypsin 4
Astacin 1b	Cathepsin L3	Astacin 5	Aminopeptidase N	Carboxypeptidase E
Astacin 2	Cathepsin L8	Astacin 7	Glutamyl aminopeptidase (3)	Triacylglycerol lipase 1
Astacin 21	Legumain 1	Astacin 9	Probable carboxypeptidase PM20D1	Triacylglycerol lipase 4
Astacin 22	Alpha-aspartyl dipeptidase	CUB/LDL trypsin 2	Peptidase M20 domain-containing protein 2 (2)	Deoxyribonuclease
Astacin 3	Probable serine carboxypeptidase CPVL	CUB/LDL trypsin 3	Methionine aminopeptidase 2	
Astacin 30	Dipeptidyl peptidase 1	CUB/LDL trypsin 5	Group XV phospholipase A2	Digestive Fluid and Fed (1)
Astacin 31	Dipeptidyl peptidase 3	CUB/LDL trypsin 6	Lysosomal alpha-mannosidase	Chitinase 2
Astacin 43	Probable aminopeptidase NPEPL1	CUB/LDL trypsin 7	Mannosyl-oligosaccharide alpha-1,2-mannosidase isoform A	
Astacin 41	Putative aminopeptidase W07G4.4	CUB/LDL trypsin 8	Maltase-glucoamylase	
Astacin 8	Alpha-L-fucosidase	carboxypeptidase B 2	Lysosomal alpha-glucosidase (2)	
Astacin 28	Alpha-N-acetylglucosaminidase	carboxypeptidase B 3	Alpha-galactosidase A	
Cathepsin L2	Neutral alpha-glucosidase AB	Lysosomal Alpha-glucosidase		
Cathepsin B1	Putative Phospholipase B-like 2	Alpha-amylase		
Cathepsin F		Alpha-N-acetylgalactosaminidase		
CUB/LDL trypsin 1		Chitinase 3		
Aspartic peptidase 1		Triacylglycerol lipase 2		
Zinc metallopeptidase		Triacylglycerol lipase 3		
carboxypeptidase B 1		Pancreatic triacylglycerol lipase		
Lysosomal protective protein		Phospholipase A2		
Cytosol aminopeptidase				
lysosomal alpha-mannosidase				
Beta-Hexosaminidase subunits alpha/ beta				
Beta galactosidase				
Chitinase

### Label-free quantitative analysis

The DF samples were individually analyzed after different periods of feeding. In general, no significant differences were detected among the protein composition/quantity of these samples (Additional file 6). Thus, the data set was treated all together. The selected digestive enzymes (Table 1) present in the digestive fluid, fasting and fed spiders represent, respectively, 39, 7 and 4 % of the total normalized spectral abundance factor (NSAF) analysis. Curiously, unknown proteins amount to 28, 17 and 6 % in the DF, and MD of fasting and fed animals NSAF, respectively.

**Fig. 3:**
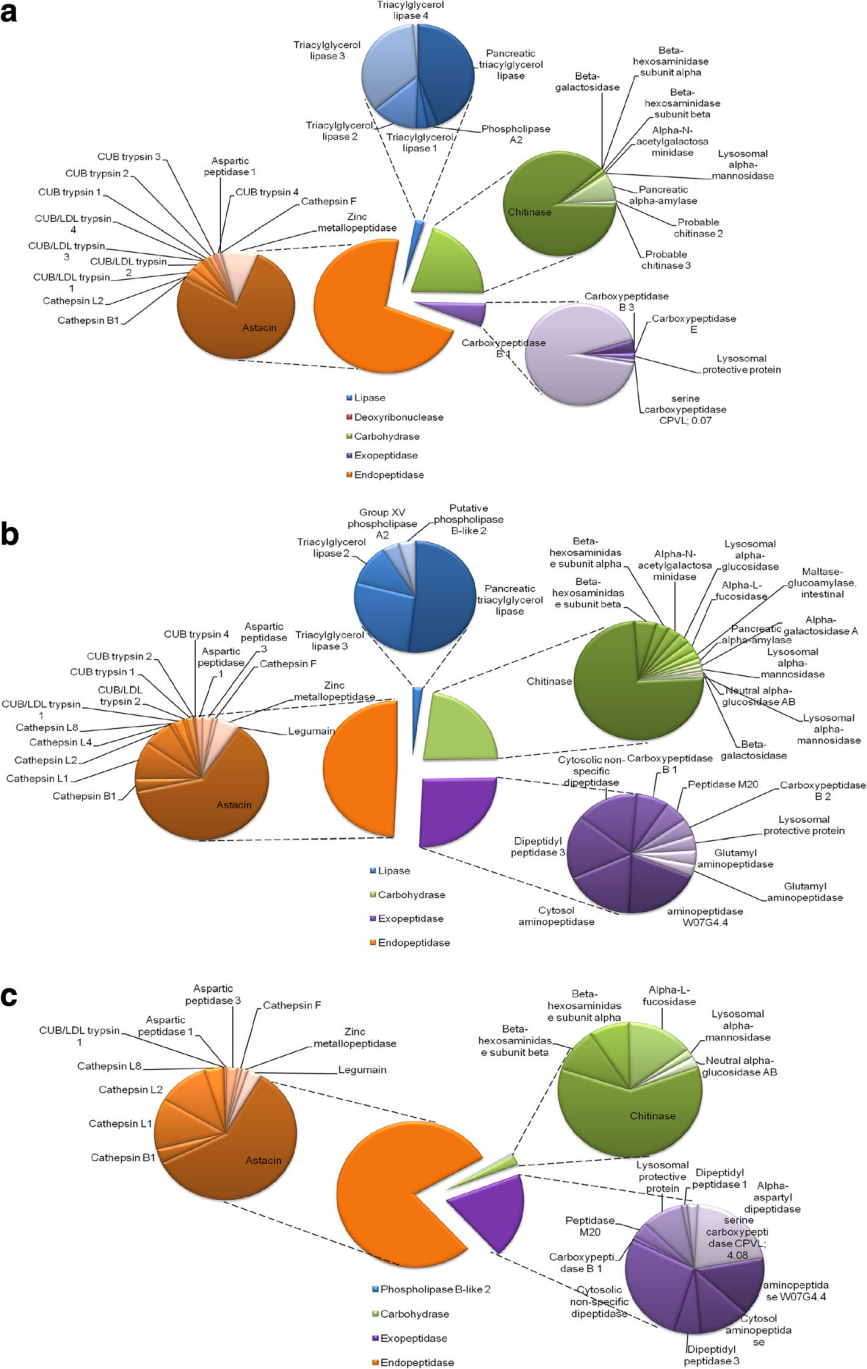
Proteome quantification using normalized spectral abundance factor (NSAF). Values represent mean percentages of digestive enzymes using 3 distinct biological replicates for midgut diverticula samples. For comparison, percentages were calculated relative only to digestive enzymes from Table 1. In DF 17 different samples were analyzed all together. **a** Fed. **b** Fasting. **c** DF. In fasting sample analysis only carbohydrases and exopeptidases with NSAF values equal or greater than 0.5 % are shown (for optimal visualization)

After these analyses we ‘normalized’ the possible digestive enzymes (Table 1) present in each sample to 100 % (Fig. 3) in order to evaluate each hydrolase group. Peptidases, carbohydrases and lipases are respectively 78, 20 and 2 % of the digestive enzymes in the DF whereas in fasting samples they represent 75, 23 and 2 %. In the MD of fed animals mainly peptidases were identified (97 %). Chitinase is quantitatively the most abundant protein in the DF and in the MD of fasting animals corresponding to 17 and 18 % of the NSAF, respectively. Other important identified carbohydrase in the DF is alpha-amylase (2 %). Lipases, in general, were the less represented digestive enzymes. In fasting samples, 3 triacylglycerol lipases (TAGL) and 2 phospholipases were found comprising 2 % whereas in the DF 5 TAGLs and 1 phospholipase A2 makes up 2 %.

Proteolytic enzymes are the largest class of hydrolases present in DF, fasting and fed animals (Fig. 3). Carboxypeptidase B1 is the most abundant exopeptidase in the DF with 5 % of the NSAF. However, the endopeptidases are in the spotlight. In summary, the endopeptidase sequences represent 72, 49 and 78 % of proteins identified in DF, fasting and fed animals, respectively. DF appears to harbor mainly alkaline peptidases, composed of astacins (56 %) and trypsins (9 %). As we thus far lack biochemical evidence to correctly classify these enzymes as true trypsins or chymotrypsin, we will keep their designation in this paper as “trypsins”. Fasting samples exhibit a total of 31 % of astacins and 13 % of acidic peptidases represented by cathepsins L, B, F and a single aspartic peptidase (Fig. 3b). Fed animals show a distinct proteolytic scenario with the acidic peptidases now comprising 32 % of the digestive enzymes, mainly represented by cathepsin L enzymes but also presenting legumain and cathepsins B and F sequences (Fig. 3c). Although astacins are still quantitatively the most abundant peptidases in the MD of fed spiders, fewer sequences could be identified (Table 1).

The cathepsins L are more abundant in the MD tissue, summing 24 % in fed and 9 % in fasting spiders. In contrast to that, this protein is only 0.09 % of the digestive enzymes from the DF. Oppositely to cathepsin L, trypsin-like serine endopeptidases seem to be more important in the DF compared to the MD tissue. They represent 8.5 % of the digestive enzymes from the DF with 8 different isoforms. In fasting animals they represent 3 % with 5 identified isoforms. Yet, in fed animals only a single one was identified (0.2 %).

### Differential expression analysis of the transcriptome and proteome data

Our transcriptome data indicate distinct expression of some hydrolases in fed and fasting animals. Histological evidence shows that the re-synthesis of digestive enzymes is a process initiated rapidly after prey capture [4]. In order to understand differential expression, analyses of the transcriptome and proteome data were performed in fasting animals and with distinct feeding periods in order to pinpoint the genes which are up and down-regulated. Comparison of 1 h fed spiders versus fasting spiders samples retrieved 48 up and 12 down-regulated genes (Additional file 7). Curiously, among the up-regulated ones, 20 sequences in the 1-h fed sample and 9 sequences in the fasting samples were non-identified contigs. The results obtained from the comparison of the 9 h fed animals versus fasting spiders are that 169 genes are overexpressed while 113 are underexpressed, with respectively 69 and 39 unidentified sequences. A large number of distinctly expressed genes were also observed in the comparison of the 9 h fed versus 1 h after feeding resulting in 262 up and 219 down-regulated genes. The enrichment analyses of both up and down-regulated contigs, based on the GO terms using the reference transcriptome indicate that the down-regulated expressed genes did not yield statistical significance in the enrichment analysis. However, this analysis for the up-regulated contigs resulted in statistically significant differential expression (Additional file 8).

Although in the 1 h fed samples versus the fasting samples comparison the number of differentially expressed genes was lower than the observed in the comparisons of other samples, the enrichment analysis showed more clearly the differences considering the GO terms. GO terms of the up-regulated genes after 1 h feeding are all clearly related to the feeding stimulus (Additional file 8). The comparison of gene expression between an animal that was exposed to food for 9 h and the ones in fasting conditions suggests that the up-regulated genes in this condition are not only exclusively involved with diet hydrolysis but that they are also involved with other processes including cellular and organism development and cell differentiation (Additional file 8). The increase of expression observed in actin binding proteins and also other proteins related to cell structure are probably related to the organization needed for the vesicular trafficking (Additional file 8 and Table 2).

**Table 2:** Genes differentially expressed in the three physiological conditions: fasting, 1 and 9 h fed animals

9 h vs 1 h		
Up regulated	log_2_ Fold Change	*p*-value
Vesicle-fusing ATPase 1	0.78	0.00321
Carboxypeptidase E	0.85	0.00204
Signal peptidase complex catalytic subunit SEC11A	0.86	0.00015
Clathrin heavy chain 2	0.92	0.00224
Ras-specific guanine nucleotide-releasing factor RalGPS1	1.21	0.00014
Astacin-like metallopeptidase 35	1.31	0.00122
Down regulated	log_2_ Fold Change	*p*-value
Lysosomal-trafficking regulator	−1.28	0.00016
Cathepsin L-like cysteine peptidase 4	−1.25	0.00018
Leucine-rich repeat-containing protein 58	−0.90	0.00288
9 h vs Fasting		
Up regulated	log_2_ Fold Change	*p*-value
CUB domain-containing trypsin-like serine peptidase 4	1.08	0.00105
Chitotriosidase	1.18	0.00003
Astacin-like metallopeptidase 22	1.19	0.00039
Astacin-like metallopeptidase 42	1.20	0.00040
Carboxypeptidase B 1	1.22	0.00001
Carboxypeptidase E	1.32	0.00000002
Ras-specific guanine nucleotide-releasing factor RalGPS1	1.40	0.00015
Vesicle-fusing ATPase 1	1.40	0.00010
Astacin-like metallopeptidase 19a	1.56	0.00043
Astacin-like metallopeptidase 9	1.60	0.00009
Ras-related protein ced-10	1.62	0.0000002
Down regulated	log_2_ Fold Change	*p*-value
Lysosomal-trafficking regulator	−1.34	0.00058
Rab-3A-interacting protein	−1.24	0.00004
1 h vs Fasting		
Up regulated	log_2_ Fold Change	*p*-value
CUB domain-containing trypsin-like serine peptidase 2	0.96	0.00002
Astacin-like metallopeptidase 23	1.04	0.00000
Astacin-like metallopeptidase 42	1.05	0.00002
Astacin-like metallopeptidase 33	1.30	0.00002
Carboxypeptidase B 1	1.34	0.00000
Leucine-rich repeat-containing protein 15	1.38	0.00002
Biotinidase	1.68	0.00001
Astacin-like metallopeptidase 9	1.76	0.0000002

The comparison of 1 h feeding to fasting samples displayed the up-regulation of 4 astacins (namely astacin 9, 33, 42 and 46), one trypsin, one carboxypeptidase B1 and one leucine-rich repeat molecule (Table 2). No digestive enzyme or related protein is down-regulated. In the 9 h versus fasting samples comparison 7 digestive enzymes are up-regulated, including astacins, trypsin, carboxypeptidases and chitinase. Two Ras proteins and one vesicle-fusing ATPase were also observed to be upregulated in this comparison, whereas the lysosomal-trafficking regulator and Rab 3A are down-regulated (Table 2). When both fed conditions are compared, only carboxypeptidase E and astacin 35 are up-regulated in the 9 h fed sample. However, proteins involved in the vesicular trafficking and protein processing prior to vesicular targeting (signal peptidase complex catalytic subunit SEC11A) were up-regulated. Down-regulated identified contigs included lysosomal-trafficking regulator, cathepsin L 4 and a leucine-rich repeat-containing molecule.

At the protein level it was not possible to detect up-regulated enzymes after 9 h feeding against fasting animals, only down-regulated proteins were observed (Table 3). Six proteins were found in both conditions but down-regulated in the MD of fed animals and another 31 sequences were identified only in the MD of fasting animals. The majority of these enzymes were also found in the DF. This result possibly reflects the fact that, after discharging the content of the secretory vesicles into the lumen, part of these proteins is still in the prey performing EOD and could not be detected. Enrichment analysis of the DF proteome in comparison to the MD shows proteolysis, digestion and extracellular space proteins among the GO terms, which are enriched (Additional file 8).

**Table 3:** Quantitative analysis of the digestive enzymes proteome

Enzyme	Fasting (NSAF)	Fed (NSAF)	Fed/Fasting	*p*-value
Astacin-like metallopeptidase 21	1.1 ± 0.1	0.06 ± 0.006	0.055	0.001
Chitinase	16.9 ± 5.2	1.49 ± 0.619	0.08	0.007
Carboxypeptidase B 1	2 ± 0.6	0.19 ± 0.237	0.09	0.009
CUB/LDL trypsin 1	1.2 ± 0.28	0.2 ± 0.088	0.16	0.01
Lysosomal alpha-mannosidase	0.3 ± 0.1	0.05 ± 0.018	0.2	0.03
Astacin-like metallopeptidase 11	1.3 ± 0.47	0.29 ± 0.153	0.21	0.019
Group XV phospholipase A2	0.0081 ± 0.009	0	0	*
Pancreatic triacylglycerol lipase	0.093 ± 0.059	0	0	*
Triacylglycerol lipase 2	0.021 ± 0.024	0	0	*
Triacylglycerol lipase 3	0.049 ± 0.055	0	0	*
Alpha-galactosidase A	0.023 ± 0.015	0	0	*
Alpha-N-acetylgalactosaminidase	0.061 ± 0.06	0	0	*
Beta-galactosidase	0.01 ± 0.0074	0	0	*
Lysosomal alpha-glucosidase	0.057 ± 0.037	0	0	*
Lysosomal alpha-mannosidase	0.0092 ± 0.0089	0	0	*
Maltase-glucoamylase, intestinal	0.044 ± 0.062	0	0	*
Pancreatic alpha-amylase	0.029 ± 0.0022	0	0	*
Carboxypeptidase B 2	0.099 ± 0.09	0	0	*
Carboxypeptidase B 3	0.0087 ± 0.0021	0	0	*
Dipeptidyl peptidase 2	0.014 ± 0.013	0	0	*
Glutamyl aminopeptidase	0.064 ± 0.08	0	0	*
Glutamyl aminopeptidase	0.084 ± 0.11	0	0	*
Peptidase M20 domain-containing protein 2	0.006 ± 0.0044	0	0	*
Probable carboxypeptidase PM20D1	0.0086 ± 0.01	0	0	*
Tripeptidyl-peptidase 2	0.0034 ± 0.0019	0	0	*
Astacin-like metallopeptidase 10	0.011 ± 0.0046	0	0	*
Astacin-like metallopeptidase 14	0.014 ± 0.0083	0	0	*
Astacin-like metallopeptidase 15	0.037 ± 0.011	0	0	*
Astacin-like metallopeptidase 17	0.017 ± 0.013	0	0	*
Astacin-like metallopeptidase 23	0.076 ± 0.022	0	0	*
Astacin-like metallopeptidase 7a	0.012 ± 0.0089	0	0	*
Astacin-like metallopeptidase 9	0.015 ± 0.022	0	0	*
cathepsin L-like cysteine peptidase 4	0.0063 ± 0.0036	0	0	*
CUB/LDL trypsin 2	0.034 ± 0.028	0	0	*
CUB trypsin 1	0.072 ± 0.022	0	0	*
CUB trypsin 2	0.0084 ± 0.0052	0	0	*
CUB trypsin 4	0.011 ± 0.0095	0	0	*

The values are mean ± SD of three distinct biological replicates

*Abbreviation*: *NSAF* normalized spectral abundance factor

*p-value not calculated

### Non-digestive proteins identified in *N. cruentata* DF and MD

The analysis of the non-digestive protein sequences obtained by MS experiments focused mainly in the identification of proteins in the DF, i.e. after secretion, regardless the mechanism. This work provides, for the first time, strong evidence that some genes previously considered to encode toxins exclusively present in spider venoms, are transcribed and translated in the digestive system of a spider and are secreted to compose the DF. So far 12 toxins previously associated only to spider venom were found in the MD at the protein level and 8 of them in the DF (Table 4). Among these DF toxins, some are quite abundant, e.g., venom peptide isomerase (0.5 %), 2 venom allergens contain the cysteine-rich secretory protein domain (0.7 %), and 1 ctenitoxin (1.8 %). Among the MD proteins six different ctenitoxins and 2 aranetoxins were identified. Peptidase inhibitors were identified as well. MS detected two cystatins and two serpins and only one of each is also in the DF. Two serpins B1 (annotated as leukocyte elastase inhibitor) were identified only in the DF. L-cystatin is the most abundant inhibitor in the DF presenting 1.2 % of the NSAF.

**Table 4:** Non-digestive proteins identified by mass spectrometry

Protein	Digestive Fluid (%)	Fasting (%)	Fed (%)	SPCS
Venom peptide isomerase (heavy chain)	0.53	N.I	N.I	None
Venom allergen 5	0.44	N.I	N.I	Inc
Venom allergen 5	0.22	N.I	N.I	Inc
U24-ctenitoxin-Pn1a	1.82	1.6	0.38	17–18
U24-ctenitoxin-Pn1a	0.11	N.I	N.I	22–23
U24-ctenitoxin-Pn1a	0.09	0.06	0.35	17–18
U24-ctenitoxin-Pn1a	0.08	0.2	0.02	19–20
U24-ctenitoxin-Pn1a	0.04	0.08	Id	17–18
U24-ctenitoxin-Pn1a	N.I	0.05	0.22	22–23
U9-ctenitoxin-Pr1a	N.I	0.009	Id	Inc
Protease inhibitor U1-aranetoxin-Av1a	N.I	0.41	0.57	None
U3-aranetoxin-Ce1a	N.I	0.06	I	None
L-cystatin	1.2	0.26	N.I	17–18
Cystatin A2	N.I	0.7	1.16	None
Serpin B3	0.06	0.07	0.03	Inc
Serpin B6	N.I	0.03	N.I	None
Leukocyte elastase inhibitor	0.07	N.I	N.I	Inc
Leukocyte elastase inhibitor	0.003	N.I	N.I	21–22
Alpha-1 inhibitor 3	0.04	0.008	0.002	27–28
Peritrophin-1	N.I	0.16	0.74	Inc
Peritrophin-48	0.008	0.04	N.I	Inc
Transferrin	0.2	0.09	0.63	Inc
Soma ferritin	0.05	5.1	2.9	None
Soma ferritin	N.I	0.21	0.08	Inc
sum of leucine-rich repeat-containing proteins	5.9	^a^	^a^	^a^

The values are the means of the percentages from the normalized spectral abundance factor to each sample. The data from midgut diverticula are from three distinct biological replicates and only proteins identified in at least two samples were used for quantitative analysis. Digestive juice NSAF is the sum of all different periods of feeding (fasting, 1, 3, 9, 25, 30 and 48 h

*Abbreviations*: *SPCS* signal peptide cleavage site, *Inc* incomplete N-terminal sequence, *N.I* protein not identified, *Id* identified in only one replicate

^a^protein not searched in that sample or SPCS not analyzed

Transferrin and soma ferritin make up 0.25 % of the DF proteins. Five molecules contain the leucine-rich repeat domain and they represent 5.9 % of the identified proteins in the DF. A peritrophin with 6 chitin-binding domains was found in the DF in low amounts (0.008 %) and in samples from fasting animals (0.04 %). Another peritrophin was identified in both fasting and fed animals being more abundant in the latter ones.

All the complete sequences found in the DF, except for the venom peptide isomerase and soma ferritin, present a signal peptide, which is usually associated with proteins addressed to secretion or to lysosome. Some of the proteins that are exclusively identified in the MD did not have this targeting signal, with the exception of one U9-ctenitoxin-Pr1a (Table 4).

Other proteins found in the DF (Additional file 4) are: molecules involved in the immune system like 3 peptidoglycan-recognition protein, techylectin and CD109 antigen; chaperons as 78 kDa glucose-related protein, heat shock proteins 83 and 70B2; clathrin; mitochondrial enzymes (malate dehydrogenase, citrate synthase) and cytoplasmatic enzymes (isocitrate dehydrogenase).

### Phylogenetic analyses

To our knowledge this is the first report of the use of such a large number of astacins for digestion in a metazoan. *N. cruentata* astacins (NcASTs) have gone through many gene duplication events. We carried an analysis of all astacin transcripts as described in section 4.5, resulting in a total of 46 different astacin genes (paralogs). These sequences had at least 5 % differences among one another at the amino acid level, therefore minimizing the error of over-representing paralogous copies when they are in fact different isoforms derived from fewer loci. Another seven sequences had less than 5 % differences and were considered as variant of the same locus (alleles) being named not only by a number but also by “a” and “b”.

**Fig. 4:**
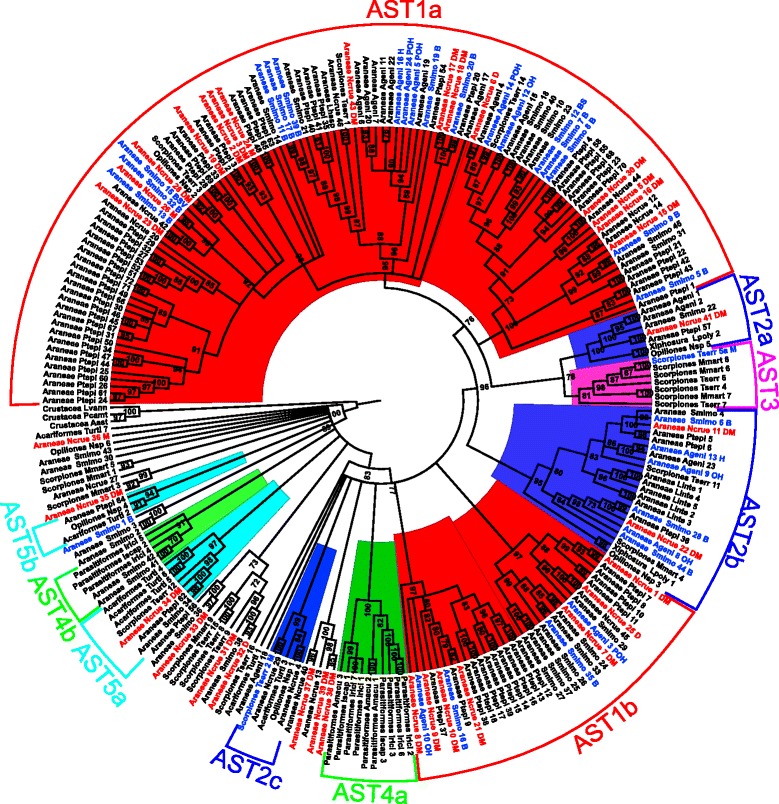
Phylogenetic analysis of arachnid astacin DNA sequences using Maximum Likelihood algorithm. UFBoot values (1000 pseudoreplicates) are shown for each node. Colored proteins in red or blue were identified by mass spectrometry in the present work or by other authors [5, 16], respectively. Letters after sequence names indicate tissue location by mass spectrometry, B, whole body; V, venom; S, silk glands; D, digestive fluid; M, midgut diverticula; O, opisthosoma; P, prosoma; H, hemolymph. Sequences were named with a number after an abbreviation used for each species as follows: Smimo, *Stegodyphus mimosarum*; Ptepi, *Parasteatoda tepidariorum*; Ncrue, *Nephilingis cruentata*; Ageni, *Acanthoscurria geniculata*; Lhesp; *Latrodectus hesperus*; Tserr, *Tityus serrulatus*; Mmart, *Mesobuthus martensii*; Iscap, *Ixodes scapularis*; Irici, *Ixodes ricinus*; Amacu, *Amblyomma maculatum*; Turti, *Tetranychus urticae*; Lpoly, *Limulus polyphemus*; Aast, *Astacus astacus*; Lvann, *Litopenaeus vannamei*; Pcamt, *Paralithodes camtschaticus*. Additional file 11 shows the accession number or contig IDs to all sequences used

The multiple alignment (Additional file 9) was quite challenging for phylogenetic analysis due to considerable variability across most of the sites. Nevertheless, this is expected due to the influence of different sources of bias within quite a large evolutionary span, such as heterogeneous levels of positive and negative selection associated with biochemical properties among sites and branches, saturation of the phylogenetic signal, inclusion of paralogous sequences, and possible missing data artifacts, among others. We have also faced a similar issue when performing phylogenetic analyses of cathepsin L sequences [16]. After the multiple alignment, the maximum likelihood (ML) algorithm was used to obtain the evolutionary history of this Arachnida gene family (Fig. 4, Additional file 10), (http://dx.doi.org/10.5061/dryad.b34s4).

**Fig. 5:**
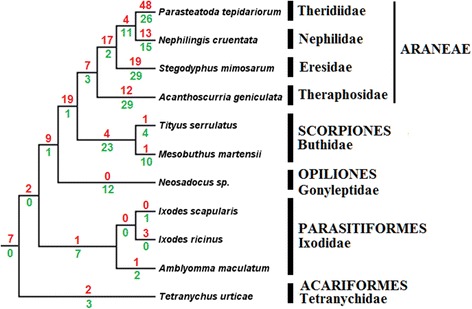
Reconciliation of ML gene tree with species tree according to [17]. Red and green numbers in each branch respectively represents duplication and losses

Gene duplications and losses were estimated (Fig. 5) using the cost algorithm within Notung (with default options, duplications =1.0 and losses = 1.5), by assuming the ML gene tree is evolving within a species tree; for the latter, we assumed the Arachnida species tree based on a set of 3644 loci, which is the most inclusive dataset analyzed so far [17] (Figures two and eight in the referred paper).

We emphasize that Notung’s algorithm of minimizing duplications and losses throughout the tree is currently not able to account for events of convergent evolution or other sources of homoplasy in orthologs, which may be the case for some of the inferred duplications (such as the presence of scorpion’s copies within AST1a), and therefore the values reported on top of branches and below them in Fig. 5 (respectively gain and losses) may be overestimated. Other issues that may contribute to this overestimation are the facts that our transcriptomes (*T. serrulatus, N. cruentata, Neosadocus sp*.) are tissue-specific, lacking astacin genes expressed somewhere else, and perhaps the RNA-seq did not cover all transcripts in the studied midgut. Furthermore, there is also a lack of NGS assembled data from groups such as Amblypygi, Schizomida, Telyphonida, Pseudoscorpiones, Ricinulei and Paplipigradi, and therefore the results are interpreted here within the context of genomes from three spiders (*Acanthoscurria geniculata*, *Stegodyphus mimosarum*, *Parasteatoda tepidariorum*), one scorpion (*Mesobuthus martensii*), two ticks (*Ixodes ricinus*, *Ixodes scapularis*) and one mite (*Tetranychus urticae*). Moreover, a transcriptome from the midgut and midgut glands of the scorpion *Tityus serrulatus* is available, and we used our unpublished transcriptome data from the digestive system of the harvestman *Neosadocus sp.*. Considering the available data, the tree clearly shows that Araneae presents the largest number of duplications compared to other included groups (Fig. 5).

Apparently animals which do not need to liquefy the food externally, such as the mite *T. urticae* and the Ixodidae ticks, did not go through this large set of duplications. Sequences from Parasitiformes tended to cluster into a separate clade shown as Ast4a (Fig. 4), with only a few other sequences grouped with velvet spider astacins (Ast4b). In contrast to that, astacins from Acariformes did not form a completely isolated group, being clustered with Araneae, Opiliones and Scorpiones (Fig. 4, AST2c, AST5a and AST5b).

Spider astacins accumulated a huge number of duplications (Fig. 5) and two general observations can be made regarding this feature. First, the number of duplications is larger in araneomorph than in mygalomorph spiders, the latter spanning from a more basal node in the Araneae species tree. Second, even though some duplications are shared among the different spider species, each one has a large number of specific paralogs as well (Fig. 5). Therefore, whilst the spider ancestor had already more astacin duplications than other arachnids, this duplication process is likely an ongoing process in spiders. The group AST1a is formed almost exclusively by spider astacins, yet three scorpion’s and one harvestman’s astacin also clustered within this group (Fig. 4). In this clade it is possible to observe that each subgroup is formed majorly by one of the spiders, highlighting the above mentioned fact of specific paralogs. The same is observed in AST1b and AST1c which are solely composed of spider astacins. Even though there were no groups within the sampled taxa that corresponded to the species tree, in clades AST2a, AST2b and AST2c it is possible to observe clustering with other arachnid orders like Xiphosura, Scorpiones, Acariformes and Opiliones. A group formed by scorpion astacins (AST3) included a copy likely associated with digestion in *T. serrulatus*, and the tree showed that scorpion sequences frequently are found next to spider astacins, sometimes even with other orders being included in the same well supported branch. This proximity of spiders and scorpions resembles the species tree of Sharma et. al (2014), which was also the species tree we used to assess duplications in the ML gene tree.

Regarding function, we believe it is not trivial to associate it to obtained clades even if more information (e.g., tissue location, actual translation) was available. For instance, venom astacins from *Loxosceles intermedia* and *S. mimosarum* did not cluster together, with the copies of the former being a sister group to astacins identified in different tissues in the investigated spiders, while the latter (Smimo 15) was also identified in the animal silk glands and hemolymph, clustering in a subclade within AST1a together with the digestive NcAst 23, non-secreted NcAst26, and two other enzymes from the velvet spider obtained from the whole body.

## Discussion

### General analysis of transcriptome and proteome data

The data set obtained in this study is the first specific massive sequencing at both mRNA and protein levels of the isolated opisthosomal midgut with its diverticula and digestive fluid of a spider. About one third of *de novo* assembled contigs presented similarity with known proteins from the databases while approximately 90 % of the proteins identified by MS were similar to sequences on databases. Although two spider genomes were recently described [5] these authors focused on the analysis of silk and venom composition. Thus, efforts on sequencing specific tissues are still needed to fully understand the distinct physiological systems. The use of these techniques in samples from the MD of the spider *N. cruentata* allowed the identification of the secreted proteins in its DF, representing the first molecular evidence that corroborates the previous histological observation [4] that the secretory cells contain the secreted digestive enzymes. Furthermore, some proteins were identified in both DF secretion and MD tissue (Tables 1 and 4, Additional file 4).

### Physiology of digestion in *N. cruentata*

**Fig. 6:**
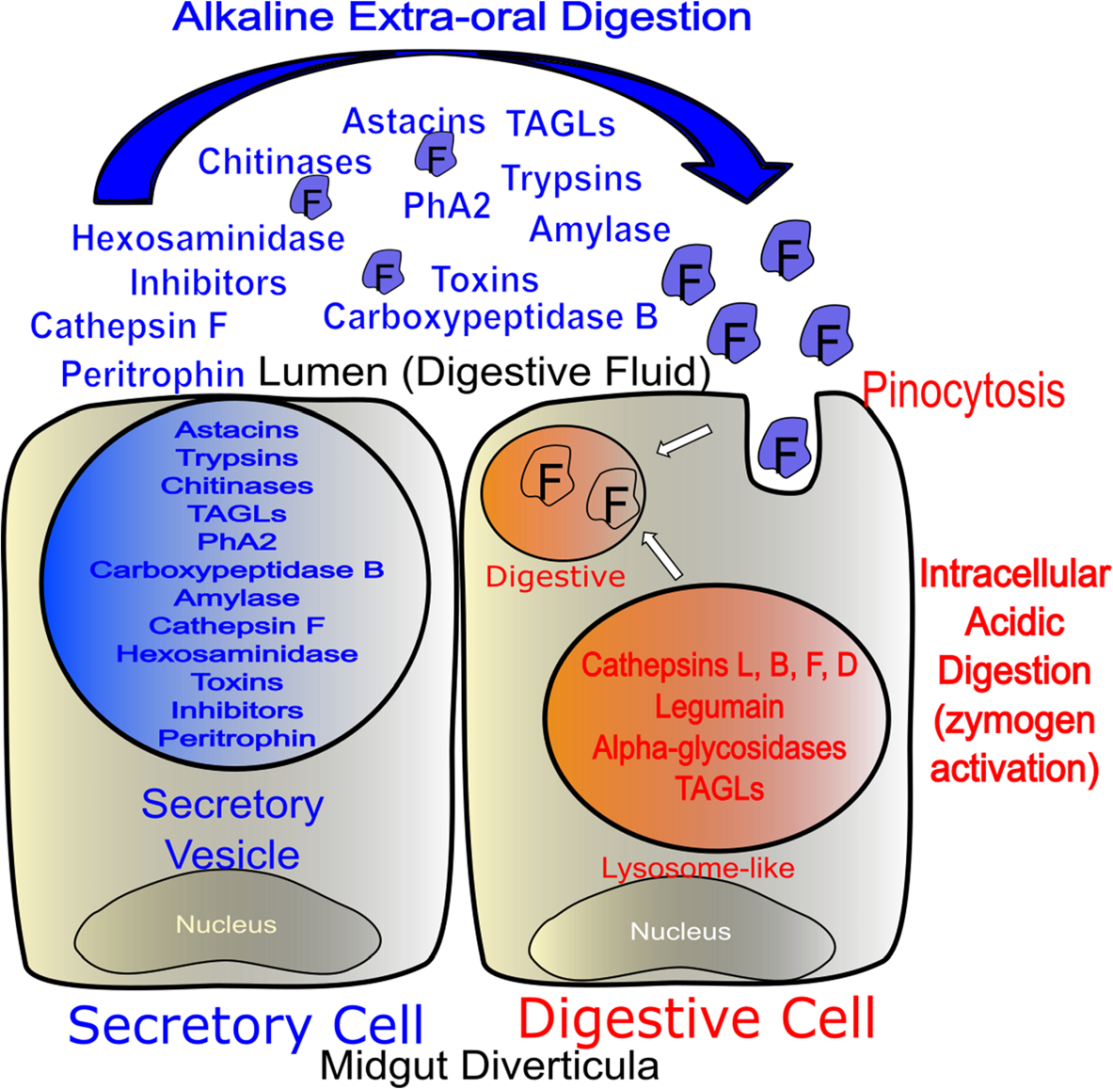
Schematic representation of digestive and secretory cells present in MD of *N. cruentata*. The content of the secretory vesicles present in secretory cells represents the DF. F: partially digested food; PhA2: phospholipase A2

Both, MD tissue and DF, are prepared prior to the next predation event, confirming the previous histological observation that secretory granules are filled/stocked up in fasting animals [4]. In conclusion, spiders already contain some DF in the midgut lumen during starvation. However, this DF will be enhanced by the discharge of the secretory granules into the lumen after prey capture. Quickly after the discharge of secretory vesicles, cells restart protein synthesis and secretion vesicle formation in preparation to the next predation event [4] (Fig. 6). These secretory vesicles contain astacins and trypsins as the main endopeptidases involved in prey liquefying but cysteine cathepsins are also present and may be required in cases of a more acidic extracellular digestion. Astacins probably act as multi-associated molecules (the same or different isoforms), which would increase their catalytic specificity and efficiency. Carboxypeptidase B1 is quite abundant (Fig. 3a) and it could be related to the degradation of astacin and trypsin products.

Regarding the carbohydrate hydrolysis machinery present in the secreted vesicles, chitinase is the most representative enzyme in the DF and this is likely related to the fact that arthropods are the most common spider preys, with their cuticular exoskeleton being the first barrier to be overcome by the EOD enzymes. The digestion of the chitin products will be performed by beta-N-acetylglucosaminidase and another hexosaminidase [12] during both digestion phases outside or inside the cell. The hydrolysis of α 1,4 linked carbohydrates is processed by the pair α-amylase/ α-glucosidase. However, they are compartmentalized in the two distinct phases of digestion, as only amylase is present in the secretory vesicles. The products of amylase hydrolysis are probably absorbed by pinocytosis in the digestive cells and then processed by α-glucosidase. Finally, triacylglycerol lipases were also identified as a component of the secretory vesicles being involved in EOD.

After internalization of the partially digested food, other enzymes become more important. Mainly acidic enzymes as cathepsins L 1, 2, 4 and 8, cathepsin D 1, cathepsin F, cathepsin B 1 and legumain 1 will accomplish the initial protein intracellular digestion. Our data indicate that maybe some astacins, as astacin 26 and 36, still have a digestive role inside the cell and are not secreted (Table 1). Final protein digestion will be performed by dipeptidyl peptidase 1, 2 and 3, serine carboxypeptidase CPVL, alpha-aspartyl dipeptidase, carboxypeptidase B 1 and 2, glutamyl aminopeptidase and cathepsin B1. For the carbohydrase intracellular digestion possible enzymes involved are: alpha-L-fucosidase 1 and 2, maltase-glucoamylase, alpha-glucosidase, alpha and beta-galactosidases, alpha and beta-mannosidases. Lipid intracellular digestion may be done by TAGL and phospholipases.

In differential expression analysis of the transcriptome data, few enzymes and proteins related to the digestive process were found differentially expressed and they were mainly found when fed animals (nine and 1 h) expression patterns were compared with the fasting ones. Basically, all possible digestive enzymes over-expressed in this comparison are related to extracellular digestion (Table 2). These differences of expression should still be validated by quantitative PCR, but these analyses indicated possible candidates. In contrast to the up-regulation visualized through the mRNA level coding for enzymes involved in extracellular digestion, at the protein level it was possible to detect only down-regulated proteins after 9 h of feeding (Table 2). At this point of feeding, it seems that the secretory vesicles are still not completely re-synthesized or we would observe similar amount of proteins in relation to fasting animals. Also, the fact that secreted enzymes were virtually absent from the proteome of fed spiders may have two possible not exclusive explanations: 1) part of the enzymes are still outside the body, liquefying the prey, and/or are in spider faeces 2) the secreted enzymes are kept in the lumen complexed with food under digestion and have not made their way back into the cells. This could be achieved through the presence of the peritrophins and the constitution of a peritrophic gel or membrane, which compartmentalizes the digestive process. In this case, these enzymes could have been lost in the centrifugation step. Histological observations demonstrated that part of the digestive vacuoles remain intact for long periods [4]. Upon re-entry into the midgut cells, the digestive enzymes would probably become detectable by our proteomic analysis, which does not seem to be happening. A study of spider faeces would be necessary to completely analyze this aspect of the digestive process. The DF composition does not seem to pass through significant changes during the feeding process as shown in Additional file 6, indicating that after the unique release of the secretory granules contents all enzymes needed for EOD are present in the DF during the entire cycle.

Furthermore, many different signaling molecules were sequenced in the DF, indicating a more complex function than only digestion. The biochemical characterization of DF previously done by other authors could be correlated to the enzymes identified in the present study. Besides that, the study of some already known proteins, the presence of a high quantity of unidentified proteins and proteins with unknown function in the DF, opens the door to a huge source of natural active biomolecules that could be tested in a range of uses, from industrial biotechnology to disease treatment besides the comprehension of spider physiology and evolution.

### Corroborating historical biochemical data with “-omics” analyses

#### Peptidases

In a series of four different articles, Mommsen was among the first investigators to do a plain biochemical characterization of many hydrolase activities in spider DF [9, 11–13]. All peptidase activities previously identified could be associated with the *N. cruentata* proteins identified by MS in the present study, excepted for the lack of aminopeptidase. Mommsen [9] reported that carboxypeptidase A was one of the most active hydrolases in the DF of *Tegenaria atrica*. Carboxypeptidase B1 is among the most abundant enzymes (5.2 %) in the DF of *N. cruentata* (Fig. 3a, Table 1). Probably they are the same type of enzyme since they both belong to the subfamily M14A and BLAST searches showed that carboxypeptidase B1 presents high similarities with both carboxypeptidases A and B.

The hydrolase named by Mommsen as “protease” did not display tryptic, catheptic or peptic activity. Thereby, the only correlation possible between *T. atrica* biochemical data and proteomic data from *N. cruentata* MD is the metallopeptidase astacin. Probably the protease activity data obtained by Mommsen [9] is a result of activity of a mixture of distinct astacins due to some evidence like: 1) the molecular mass was in a range of 25 kDa which is the typical molecular mass of the astacins sequenced from *N. cruentata* DF 2) the broad pH range of activity (pHs 5–10) using azocasein as substrate is indicative that more than one enzyme was present in the DF sample used by Mommsen [9]; 3) studies with *N. cruentata* activity using a combination of casein-FITC as substrate and distinct peptidase inhibitors indicated that peptidases at these samples hydrolyzing casein are astacins (unpublished observations). Proteolytic activity using collagen as substrates has also been described in the DF and MD from spiders. Although these activities have been assigned as collagenase in the literature, they were merely hydrolases which activity was tested using collagen as substrate without further characterization [6, 7, 18]. Collagenase is a metallopeptidase from the M10A subfamily and such type of protein was identified in the present work only at the mRNA level (Additional file 1). In fact, the spider digestive enzymes named by Kavanagh and Tillinghast [8] and by Atkinson and Wright [18] as collagenase present characteristics similar to astacins. Foradori and co-workers [6] had also observed activity over casein with low molecular masses. The proteins of 16 and 18 kDa had their amino-terminal sequences determined. These enzymes presented high similarity to the astacin 19 identified in the DF of *N. cruentata*. These authors had also observed that astacins form oligomers [6]. Such association was also observed for other arthropods [19].

Our study clearly shows the dependence of astacins in EOD process in an orb-weaver spider, since astacins in the DF makes up 56 % of the digestive enzymes. Astacin is also the most abundant hydrolase in the MD (Fig. 3a and b). The proteome results from Sangaard [5] identified 19 and 10 astacins in the “whole body” of the velvet spider *S. mimosarum* and tarantula *Acanthoscurria geniculata*, respectively. Although the experiment was not tissue specific, those values are close to the number of astacins used for digestion by *N. cruentata* and reinforces the importance of astacins in spiders.

The scorpion *Tityus serrulatus* has two digestive astacins [16]. Ticks rely in their digestive process mainly on the activity of intracellular acidic peptidases such as cathepsins L, B and D [20] due to their particular feeding habits in contrast to other arachnid species, but they also present two astacins. It seems reasonable to hypothesize, with the available Arachnida data available till now, that in evolution astacins became more important for EOD in Araneae, with subsequent gene duplication events in this group, since araneomorph spiders contain almost double the number of astacins compared to mygalomorph ones.

Besides astacin other endopeptidases are present in the MD of spiders. Foradori and collaborators [7] observed the cleavage of collagen, fribrinogen, fibrin, fibronectin and elastin in the DF of *A. aurantia*. Only 65 % inhibition was observed using EDTA which indicates the presence of other alkaline peptidases such as trypsins and chymotrypsins. Two chymotrypsins- and 2 trypsins-like activities were identified in *T. atrica* DF while 8 proteins with a trypsin/chymotrypsin domain were found in the digestive juice of *N. cruentata*. Curiously, all trypsin/chymotrypsin found in *N. cruentata* at the protein level present CUB (complement protein C1r/C1s, uEGF and Bmp1) and Low Density Lipoprotein receptor (LDLR) domains, distinctly of other arthropod digestive trypsin/chymotrypsins, which usually only have the catalytic domain [21, 22]. Receptor mediated endocytosis is a possible function of these domains since the pinocytic activity during prey digestion is significantly increased. Serine peptidase/trypsin proteomic sequences were obtained from the whole body of the velvet spider (6 trypsins) and the tarantula (17). The velvet spider presented 3 catalytic trypsins with CUB and LDLR domains as *Nephilingis* trypsins: 2 containing one LDLR domain and only 1 trypsin containing exclusively the catalytic domain. The tarantula presented 5 trypsins containing exclusively the catalytic domain; 3 containing CUB, LDLR and catalytic domain; 1 trypsin containing LDLR domain and 1 containing two scavenger domains (SRCR) and LDLR domain. The association of domains involved in protein: protein interaction seems essential to spider trypsins.

Acidic peptidases are present at very low abundance in the DF (Additional file 6). These cathepsins may be useful to extracellular digestion if, due to the prey tissue nature, a more accentuated acidification occurs. It was already shown that cathepsin L displays collagenolytic activity in acidic conditions [23, 24]. Our group characterized the cysteine cathepsins present in the MD of *N. cruentata*, which presented acidic characteristics such as pH optima 5 and conversion of the zymogen to the mature form after acidification [10]. Activity was not detected in the DF and cysteine cathepsins are more abundant in the MD (Fig. 3, Additional file 6). It seems that cysteine cathepsins, mainly cathepsin L, will be more important to the acidic intracellular digestion in contrast to the majority of astacins and trypsins used for EOD.

#### Carbohydrases, lipases and nucleases

Transcriptomic and proteomic data from *N. cruentata* presented in this work corroborate the previously described analysis of the carbohydrases from the DF of the spiders *T. atrica* and *Cuppienius salei* [11, 12, 25]*.* The most active carbohydrase identified in the DF of *T. atrica* [11] and *C. salei* [12] is one chitinase. Chitinase is the most abundant (18 % of the digestive enzymes and comprises 7 % of all the proteins in the DF (Additional file 6). The mass of 48 kDa estimated by Mommsen [12] exactly matches the one predicted in our work. The whole digestion of prey exoskeleton chitin would be achieved with the activity of both endochitinases and β-N-acetylglucosaminidases (β-N-hexosaminidases), which are involved in the hydrolysis of *N,N*’-diacetylchitobiose. The activity of β-N-acetylglucosaminidase was characterized by Mommsen (1980) in the spiders previously mentioned. The activity and sequence of a β-N-acetylglucosaminidase were also identified in the DF of *N. cruetanta* (Fuzita et al., manuscript in preparation and the present work). We also identified 11 peptides of a β-hexosaminidase in all digestive fluid samples suggesting that at least two enzymes are involved in the hydrolysis of chitinase products. Another pair of enzymes involved in initial and final digestion of carbohydrates is α-amylase/ α-glucosidases. The alpha-amylase activity found in these spider species [11, 25] was also identified by MS in the present study and it composes 2 % of the digestive enzymes in the DF (Additional file 6). The molecular mass of 59 kDa predicted in the present study is similar to the ones described in literature [11]. The presence of amylase activity in predator animals is always intriguing. The first function that could be attributed to this enzyme in spiders is the digestion of glycogen present in the prey. However, some authors demonstrated that some spiders actively feed on pollen [26], and amylase and α-glucosidases could be employed for the complete digestion of pollen starch grains. The α-glucosidases was observed only in the proteomic and biochemical analysis of the MD (Fuzita et al., manuscript in preparation), indicating that probably α-glucosidases are mainly involved in the intracellular phase of digestion. Distinct from α-glucosidases other carbohydrases like beta-galactosidase and alpha-mannosidase were also identified by protein sequencing and activity assay in the DF of *N. cruentata* (Table 5).

**Table 5:** Enzymes obtained in this study and their relationship with the literature data

Reference	Enzyme	This work
Mommsen [8]	Carboxypeptidase A	Carboxypeptidases B
	Protease	Astacin(s)
	Chymotrypsin/trypsin	CUB and CUB/LDL Trypsins
	Aryl aminopeptidase	N.I.
Mommsen [9, 10]	Alpha-amylase	Alpha-amylase
	Chitinase	Chitotriosidase
	beta-N-acetylglucosaminidase	beta-hexosaminidase (?)
	Beta-glucuronidase	Beta-galactosidase
	Alpha-glucosidase	Alpha glucosidase
	Beta-glucosidase	N.I.
Mommsen [11]	Tributyrinase	TAGLs 1, 2, 3 and 4, pTAG
	Carboxylic esterase	TAGLs 1, 2, 3, 4, pTAG and Abhydrolase domain-containing protein 11
	Lipase	TAGs 1, 2, 3 and 4
	Desoxyribonuclease	Deoxyribonuclease
Kavanagh and Tillinghast [7]	Proteases A, B, C and D	Astacins
Atkinson and Wright [15]	“collagenase”	Astacins and trypsins
Foradori et al [6]	“collagenase”	Astacins and trypsins
Foradori et al [5]	p16 an p18	Astacin 19a

The lipases found in the DF of the spider *N. cruentata* were basically TAGLs (Table 2). Such type of enzyme is probably related to the tributyrinase, lipase and esterase activities observed by Mommsen [13] (Table 5) and observed also in *N. cruentata* DF and MD [27]. TAGLs 2, 3 and pancreatic TAG are present in both MD and DF, whereas TAGLs 1 and 4 are present only in the DF. The DNase activity in the DF of *T. atrica* can probably be attributed to the deoxyribonuclease identified in the present study.

### Evolutionary aspects of digestive process in spiders

The digestive process in spiders and most part of other predator arachnids can be considered unique among metazoans. These animals combine a mechanism of extra-corporeal prey liquefying with a subsequent completion of the digestion inside the midgut cells. In a previous work we hypothesized on evolutionary aspects of digestion in arachnids based mainly in tick, mite and scorpion data. Briefly, to these groups of arachnids, due to intracellular digestion, acidic peptidases such as cathepsin L became more important with, at least, 15 duplications observed [16]. In the present work it was observed that the peptidases used for EOD in Araneae are mainly astacins and they have also passed through many gene duplication events. The number of detected paralogs is higher for spiders than for other arachnids (Fig. 5). For instance in the midgut and midgut glands of the scorpion *T. serrulatus*, a basal arachnid, only two astacins were identified at the protein level using a similar approach [16], in contrast to 25 in the MD of *N. cruentata* (Table 1). Ticks also present only 2 astacins, which seem to be involved in digestion [28]. As we observed for cathepsin L [16], most part of Parasitiformes’ astacins did not group with other arachnid sequences (Fig. 4), probably due to specific selective pressures over the blood-feeding habits in this group. In contrast to other arachnids, during Araneae evolution astacins have duplicated, possibly acquiring different functions, and being more abundant in derived spiders (Fig. 5).

The use of astacins for meal protein hydrolysis was clear in *N. cruentata* due to its presence in the DF. However, due to the lack of biochemical and sequence tissue-specific information for other arachnid lineages won’t be reasonable extrapolate our data to other spiders/arachnids. The proteins observed in the MD and DF, which was previously only identified in other spider’s venom, may indicate the original source of toxins when more sequences became available. The increase in biochemical, tissue-specific sequences and paired analyses between venom and MD or venom and DF preferentially from the same species will allow evolutionary comparisons as the ones made for snakes [29].

In the present study, it was shown for the first time that astacin gene duplications are pervasive within the arachnid lineage, an untested hypothesis so far [5]. Both recent and old duplications were detected; relatively older ones are demonstrated by the presence of the same species lineages in different parts of the tree; and in-paralogs suggest that the digestive protein machinery is continuously evolving in different spider species probably acquiring new functions.

## Conclusion

This work is the first specific tissue massive mRNA and protein data of the digestive system and proteome of the DF of spiders. This analysis is the first molecular demonstration of the hypothesis suggested by the histological studies that DF is a secretion originating from the MD. Our data also enhanced the comprehension of the effect of feeding conditions in spider digestive system. Peptidases are one of the most representative groups of digestive enzymes identified. Phylogenetic analyses indicated an extensive gene duplication of astacin genes being an important hallmark of the digestion evolution in spiders. We were able to identify both, mRNA and proteins, of toxins believed to be exclusively expressed in venom glands. The recruitment of originally digestive genes to venom composition seems not a peculiar characteristic of spiders but it has been described to other predators as snakes [29, 30]. We identified enzymes involved in the extracellular and intracellular phases of digestion allowing the design of the first cellular/molecular model of the digestive process in spiders.

## Methods

### Animals and sample obtaining

Adult *N. cruentata* females were collected at Instituto Butantan (São Paulo, SP, Brazil) kept under natural photoregime and room temperature conditions with water spraying 4 times per week in their artificial environment. The animals were starved for at least 1 week and then fed with *Acheta domesticus*. After 1 and 9 h of unlimited access to their food, the ‘fed’ animals were dissected whereas the MD from fasting spiders was removed 2 weeks after start feeding. The dissection was performed in a cold isotonic saline solution (300 mM KCl pH 7) after anesthetizing the animals in a CO_2_ chamber and the opisthosomal midgut with its diverticula (MD) were removed. In the samples used for RNA extraction, the saline solution was made with autoclaved sterilized water containing 0.1 % (v/v) diethyl pirocarbonate (DEPC) and all dissection material was cleaned with 70 % ethanol (v/v), placed under UV light for 30 min and subsequently heated to 150 °C for 4 h. Digestive fluid (DF) samples were collected by electrical or mechanical stimulus in 2 weeks fastened or 1, 3, 9, 25 or 48 h fed spiders.

### cDNA library preparation and sequencing

All enzymes, primers and buffers cited in this section are from Illumina® unless otherwise specified. RNA extraction was done using TRIzol® reagent (Invitrogen) according the manufacturer’s instructions. The RNA amount was spectrophotometrically quantified at 260 nm and its purity evaluated by the absorbance ratio 260 nm and 280 nm. The RNA quality and integrity were analyzed in the Agilent 2100 Bioanalyzer (Agilent Technologies).

Poly-adenylated mRNA was purified with oligo (dT) magnetic beads (Illumina®) according to their standard protocol (http:/grcf.jhmi.edu/hts/protocols/mRNA-Seq_SamplePrep_1004898_D.pdf). Thereafter, cDNA was reverse transcribed and cloned. In brief, the mRNA was fragmented in the proper buffer and the first cDNA strand synthesis was made using Superscript II® Reverse Transcriptase (Invitrogen). After subsequent RNase H treatment the second cDNA strand was synthesized by DNA polymerase I. The end of the molecules were phosphorylated and the 3’ terminal adenylated using the enzymes T4 PNK and Klenow exo, respectively. The adapters were then linked to the DNA fragments with a T4 DNA ligase. After that, the libraries were amplified with primers specific to the adapters.

The quality of the library constructed was validated by the Agilent 2100 Bioanalyzer (Agilent Technologies) with the chip DNA 1000 and quantified by quantitative polymerase chain reaction with the kit KAPA Library Quantification (KAPA biosystems). The library was diluted to a final concentration of 20 pM and each one was clustered and amplified by using the TruSeq PE Cluster Kit v30cBot-HS. Next generation sequencing was performed in a HiScanSQ (Illumina®) using the TruSeq SBS Kit v3-HS (200 cycles) according to the manufacturer’s instructions.

### Computational analyses

The transcriptomic experiments were performed in triplicate for spiders that were under 3 different physiological conditions, fasting, 1 and 9 h fed. The differential expression was studied using the software DESeq 2 (http://bioconductor.org/packages/2.13/bioc/html/DESeq.html). The HiScanSq (Illumina®) data obtained were analyzed in four main steps. In the raw data obtainment step the software package CASAVA (2011) 1.8.2 (Illumina®) was employed. This algorithm makes the base call from raw data transforming them into fastq format reads followed by the phred’s quality scores. The reads were visualized with the program FastQC 0.10.1 and then the Agalma pipeline shuffles the reads and removes those with low quality (less than 30 nucleotides). Next, vectors, primers and ribosomal RNA sequences were withdrawn after comparison with the Univec and ribosomal RNA databases, both from NCBI (National Center for Biotechnology Information).

De novo assembly was done by the programs Velvet/Oases incorporated to the Agalma pipeline [31, 32]. Four assemblies were done to all samples with kmers of 31, 41, 51 and 61 that thereafter were merged and the redundant contigs removed. A BLAST (basic local alignment search tool) [33]) was used to identify and annotate assembled sequences using the UniProt as a database with an e-value threshold of 10^−10^. Fasta files were filtered by removal of transcripts smaller than 150 bp, splice variants and low confidence contigs. Additional file 1 contains the final non-redundant contig database. After de novo assembly all hydrolase sequences considered as possible digestive enzymes presented in Table 1 were manually curated in order to obtain a non-redundant number of different proteins.

The gene ontology (GO) was obtained using the program Blast2GO [34] with the non-redundant NCBI database. The e-value and annotation cutoff were respectively 10^−6^ and 45. The enrichment analysis was performed using Fisher’s exact test with multiple testing correcting of false discovery rate using the differentially expressed genes as test group against the entire transcriptome data set in the formerly cited software. The same analysis was performed with the digestive fluid proteome versus the MD proteome. The contig translation based on the DNA coding regions was performed using the software FrameDP v 1.2.0 [35]. After using the BLASTX tool against the UniProt database the program created a training set to predict the more likely coding DNA sequence (CDS) based on the interpolated Markov models (IMMs). Contigs with less than 50 amino acids were removed. Additional file 3 has the translated database used for protein identification. Signal peptides were predicted on line using SignalP (http://www.cbs.dtu.dk/services/SignalP/).

### Proteomic procedures

Sample preparation [10] and MS analyses [36] were conducted as previously described. Briefly, the MD homogenates (three distinct biological replicates) were submitted to three freeze and thaw cycles and then centrifuged for 20 min at 1000 × *g*. Supernatants (50 μg) were collected and used for proteome analyses after in-gel digestion. The identification numbers showed to each sample in Fig. 1 and section 2.2 are related to a global identification using all LC-MS/MS runs from biological replicates obtained as previously described [16], in which DF, fed MD and fasting MD were treated as different samples (but including all replicates) in the software Scaffold 4 [37]. Label-free quantitative analysis was done by using the normalized spectral abundance factor (NSAF) [38] in the software Scaffold 4 [37]. In this case, each replicate of a determined physiological condition (DF, fed or fasting MD) was individually loaded into Scaffold as a single sample, and only proteins identified in at least two biological replicates were used for quantification. Due to these facts, not all of proteins identified in the general experiment appear in the quantification list, since more peptides may be attributed to a protein in the global analysis due to the higher number of samples. In all cases positive protein identification required the presence of at least 2 sequenced peptides with a calculated false discovery rate (FDR) of 0.1 % using X!Tandem [39].

### Phylogenetic analyses

Astacin sequences from arachnids were obtained after keyword searches (astacin or zinc metallopeptidase) in public databases (www.uniprot.org and www.ncbi.nlm.nih.gov/). BLAST tool [33] was also used for searching astacin sequences from arachnids with complete genomes, using as references the astacins identified in the present study or the archetypal one from *Astacus astacus* (CAA6498.1). The same approach was used for retrieving astacin homologues from *Acanthoscurria geniculata, Stegodyphus mimosarum* [40] and *Parasteatoda tepidariorum*, and also *Mesobuthus martesii* [41], in the supporting material provided by the authors. Prior to phylogenetic analyses all sequences were scanned using Pfam (http://pfam.xfam.org/) and Interpro (http://www.ebi.ac.uk/interpro/) databases and only sequences with a hit for astacin domain were used. Since a large number of possible paralogs was obtained, a neighbor-joining analysis was performed in the sequences from each organism so redundant sequences could be removed. In order to differentiate between alleles and paralogs the p-distance among the sequences was calculated. Sequences with less than 5 % difference were considered as alleles and named by a number followed by “a” or “b”, for instance astacin 1a and astacin 1b. Sequences that were more than 5 % different were considered paralogs, for instance astacin 2 and astacin 3. Only paralogs (according to the criterion above) were included in the phylogenetic analyses.

We aligned the referred sequences anchored by codon, using Guidance v1.5 [42] with Mafft v7.29 [43] as aligner to estimate site positions with low support using 100 pseudoreplicates. For each replicate Guidance uses an alternative topology (by bootstraping the original alignment) from which a multiple alignment follows, and after running the 100 pseudoreplicates, sites below a cutoff of a column score of 0.93 (which minimizes both false positives and false negatives) in relation to the original alignment were removed from the alignment, being considered unreliable. Subsequently, sites present in three or less taxa were removed (because at least four taxa are needed for the phylogeny estimation).

Phylogenetic analysis was done by Maximum likelihood (ML) in IQTree [44] using three Crustacea sequences from different species as outgroup. Partitioning analysis and model choice were done in the same software by concomitantly estimating the best partitioning strategy among codon positions (i.e., each codon position being considered separately, altogether, or in combinations of two of the positions versus a separate position), while at the same time estimating the best model for each partition. Mixture models (which consider different matrices of distributions of rates instead of the typical single matrix of rates following gamma distribution) and + ASC (ascertainment bias correction, necessary to correct for datasets in which invariable sites are not present) models were also tested along + G (gamma distribution of rates across sites) and + I (proportion of invariable sites) during the model choice phase. A total of 1000 UFBoot pseudoreplicates [45], which has a better correlation with true probability of branch existence than the regular bootstrap [46], was used to calculate branch support. Branches with UFBoot support < = 70 were collapsed using TreeGraph2 [47].

We used Notung [48] to estimate the amount of duplications across the samples studied. It uses a user-defined species tree in which a gene tree with duplications must have evolved, and then minimizes the amount of duplications. We based our Arachnida species tree in the study of Sharma et al. [17] which is based on an inclusive set of 1,235,912 sites across 3644 loci. The gene tree was the ML tree obtained as mentioned above. Regions with unsupported branches (i.e., those with support < = 70) were considered polytomies and re-optimized under Notung, which does so by searching a topology that minimizes duplications. In the end it reported the amount of duplications per branch, permitting a discussion of levels of duplications in different lineages.

## Additional files

Additional file 1: List of contigs after *de novo* assembly of RNA-seq data. (FAS 31998 kb) Additional file 2: Parameters of individual assemblies of transcriptome data. (DOCX 14 kb) Additional file 3: Translated and annotated contig database used for proteome identification. (FAS 8034 kb) Additional file 4: Qualitative proteome data. (XLSX 131 kb) Additional file 5: Gene ontology (GO) scores of DF proteome. (PDF 860 kb) Additional file 6: Proteome quantification. (XLSX 279 kb) Additional file 7: Quantitative transcriptome data. (XLSX 182 kb) Additional file 8: Enrichment analysis of GO terms using Fisher’s exact test. (a) One hour fed versus fasting spiders (transcriptome data). (b) Nine hours fed versus fasting spiders (transcriptome data). (c) All DF samples versus total MD (fasting and fed) proteomes. (PDF 159 kb) Additional file 9: Astacin multiple alignments used for phylogenetic reconstruction. (FAS 83 kb) Additional file 10: Phylogenetic analysis of arachnid astacin DNA sequences using Maximum Likelihood algorithm. UFBoot values (1000 pseudoreplicates) are shown for each node. Letters after sequence names indicate tissue location by mass spectrometry, B, whole body; V, venom; S, silk glands; D, digestive fluid; M, midgut diverticula; O, opisthosoma; P, prosoma; H, hemolymph. (PDF 13 kb) Additional file 11: Accession number or contig identification from astacin DNA sequences used for phylogenetic analysis showed in Fig. 4 (XLSX 13 kb)

## Abbreviations

CUB: Complement protein
DF: Digestive fluid
EGF: Epidermal growth factor
EOD: Extra-oral digestion
GO: Gene ontology
LDLR: Low density lipoprotein receptor
MD: Midgut diverticula
NcASTs: *Nephilingis cruentata* astacins
NGS: Next generation sequencing
NSAF: Normalized spectral abundance factor
SRCR: Scavenger domain
TAGL: Triacylglycerol lipase
