# Supplementary material for: High throughput techniques to reveal the molecular physiology and evolution of digestion in spiders
Source: BMC Genomics. 2016 Sep 7;17(1):716. doi: 10.1186/s12864-016-3048-9 (PMC5013568; doi:10.1186/s12864-016-3048-9)
Supplement: Additional file 2: — Parameters of individual assemblies of transcriptome data. (DOCX 14 kb) [file 12864_2016_3048_MOESM2_ESM.docx]

| **Sample** | **Reads*** | **Contigs** | **N50 length** |
| --- | --- | --- | --- |
| Fasting 1 | 12052323 | 17304 | 1530 |
| Fasting 2 | 21797996 | 17288 | 1483 |
| Fasting 3 | 19936020 | 16720 | 1280 |
| 1hour-1 | 16771870 | 14044 | 1393 |
| 1 hour-2 | 24653784 | 14817 | 1385 |
| 1 hour-3 | 20839346 | 18330 | 1442 |
| 9 hours-1 | 15333222 | 13331 | 1416 |
| 9 hours-2 | 24710212 | 17489 | 1422 |
| 9 hours-3 | 11915339 | 16555 | 1329 |

**Additional file 2:** General features of the MD transcriptome data

* Reads kept after using Illumina® filtering for sequencing quality
