# Supplementary figures and images for: High throughput techniques to reveal the molecular physiology and evolution of digestion in spiders
Source: BMC Genomics. 2016 Sep 7;17(1):716. doi: 10.1186/s12864-016-3048-9 (PMC5013568; doi:10.1186/s12864-016-3048-9)

## Biological Process

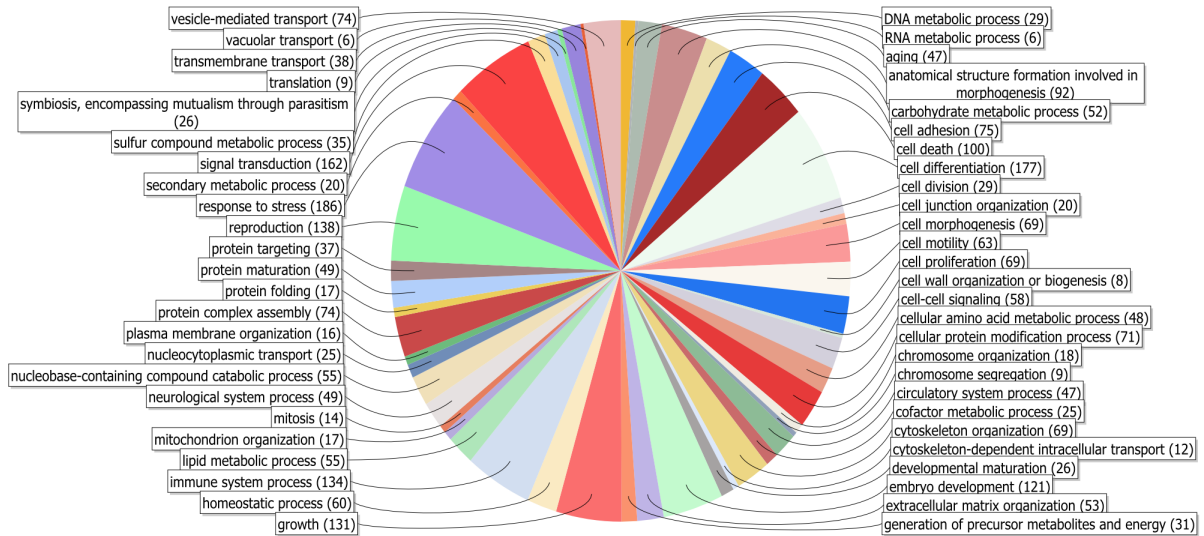

## Molecular function

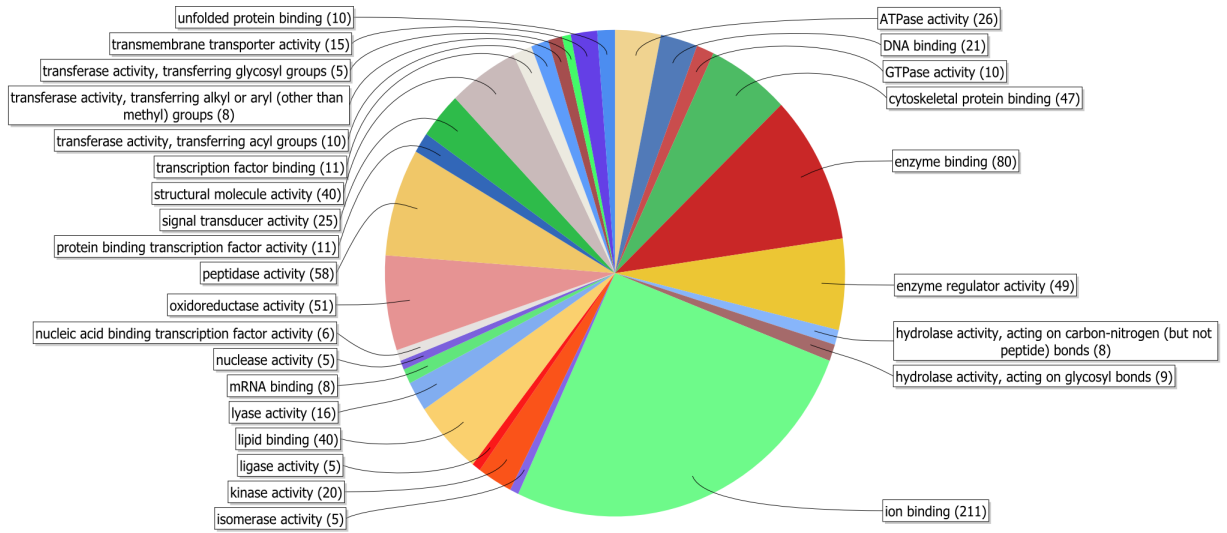

Supplement: Additional file 5: — Gene ontology (GO) scores of DF proteome. (PDF 860 kb) [file 12864_2016_3048_MOESM5_ESM.pdf]

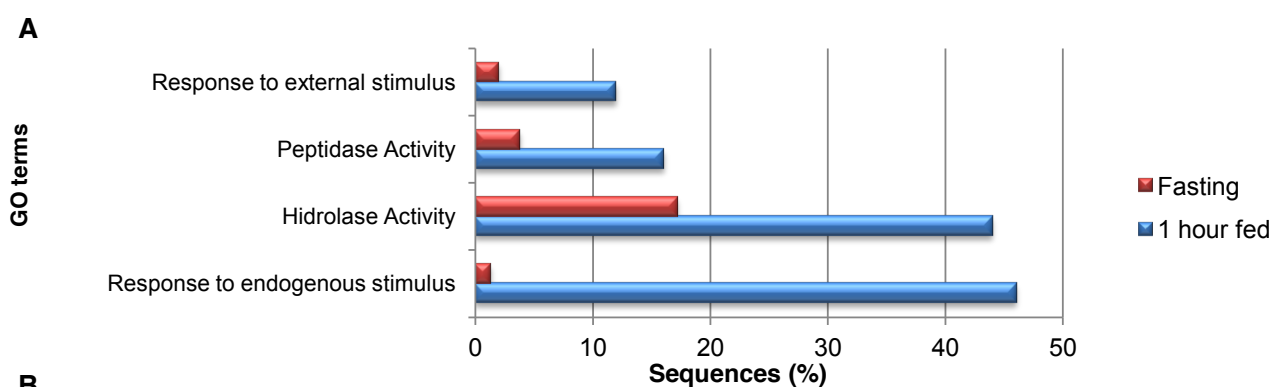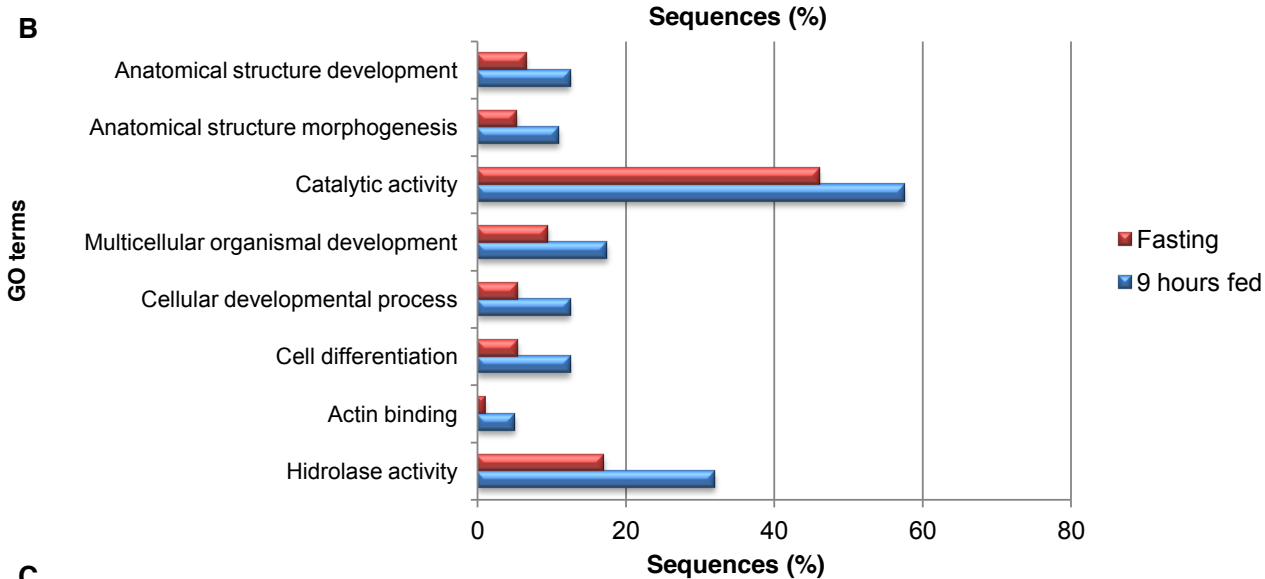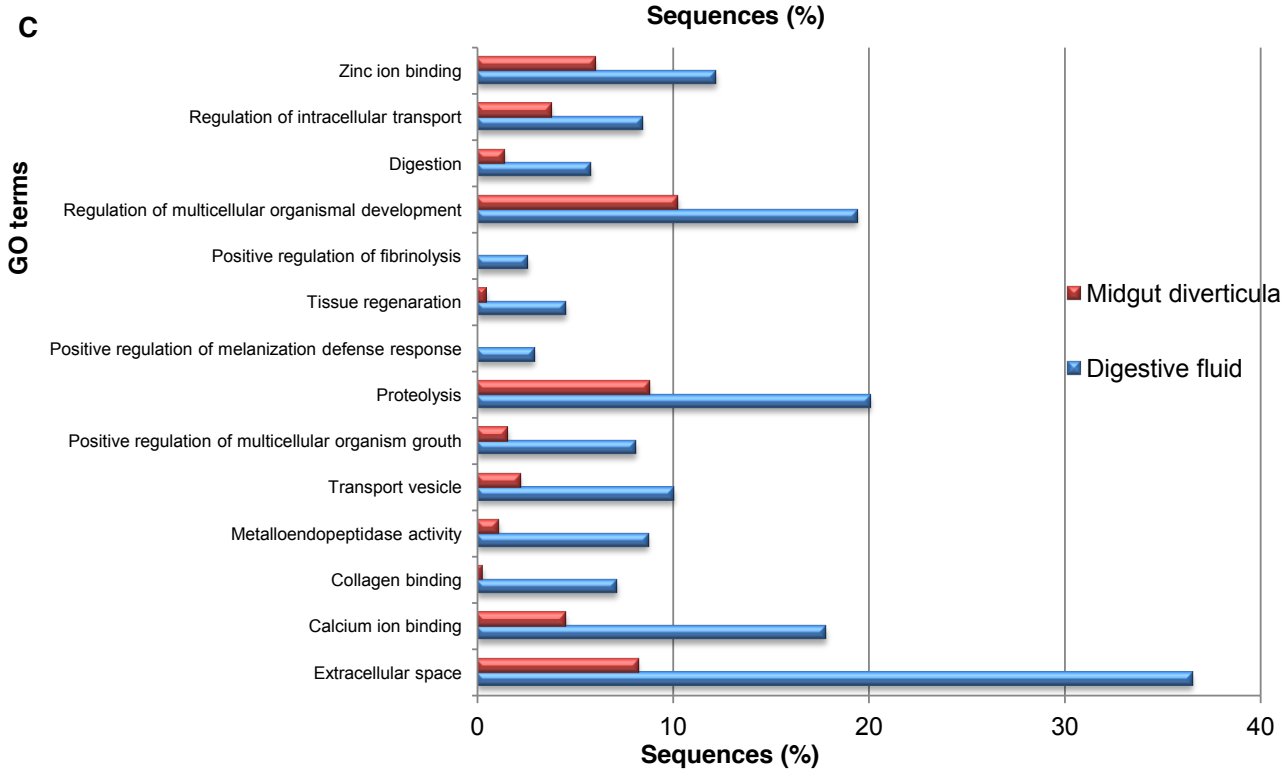

Supplement: Additional file 8: — Enrichment analysis of GO terms using Fisher’s exact test. (a) One hour fed versus fasting spiders (transcriptome data). (b) Nine hours fed versus fasting spiders (transcriptome data). (c) All DF samples versus total MD (fasting and fed) proteomes. (PDF 159 kb) [file 12864_2016_3048_MOESM8_ESM.pdf]
