# Supplementary material for: High throughput techniques to reveal the molecular physiology and evolution of digestion in spiders
Source: BMC Genomics. 2016 Sep 7;17(1):716. doi: 10.1186/s12864-016-3048-9 (PMC5013568; doi:10.1186/s12864-016-3048-9)
Supplement: Additional file 10: — Phylogenetic analysis of arachnid astacin DNA sequences using Maximum Likelihood algorithm. UFBoot values (1000 pseudoreplicates) are shown for each node. Letters after sequence names indicate tissue location by mass spectrometry, B, whole body; V, venom; S, silk glands; D, digestive fluid; M, midgut diverticula; O, opisthosoma; P, prosoma; H, hemolymph. (PDF 13 kb) [file 12864_2016_3048_MOESM10_ESM.pdf]

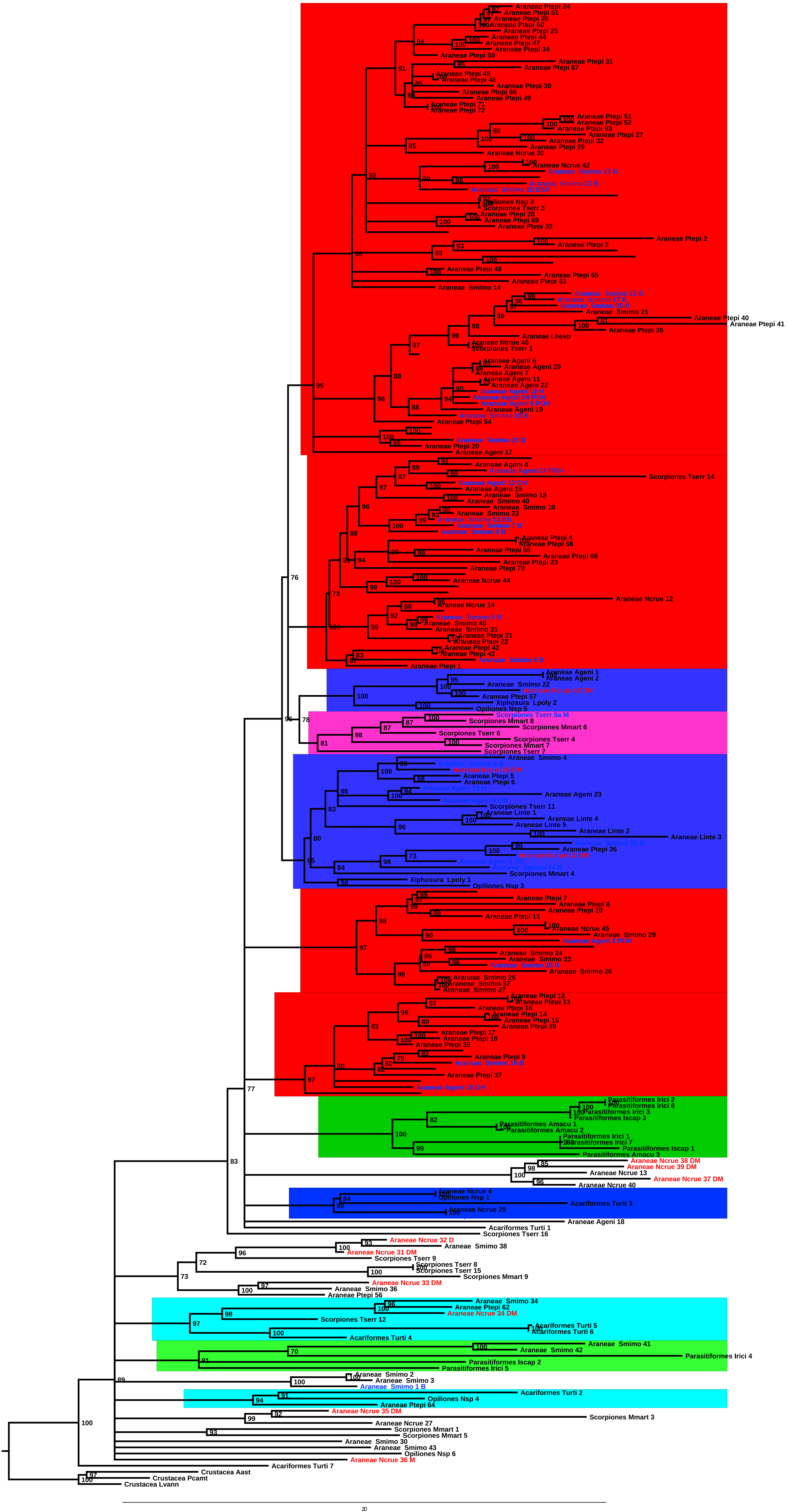
Araneae Ptepi 24
Araneae Ptepi 61
Araneae Ptepi 26
Araneae Ptepi 60
Araneae Ptepi 25
Araneae Ptepi 44
Araneae Ptepi 47
Araneae Ptepi 34
Araneae Ptepi 50
Araneae Ptepi 31
Araneae Ptepi 67
Araneae Ptepi 45
Araneae Ptepi 46
Araneae Ptepi 30
Araneae Ptepi 66
Araneae Ptepi 49
Araneae Ptepi 71
Araneae Ptepi 72
Araneae Ptepi 51
Araneae Ptepi 52
Araneae Ptepi 53
Araneae Ptepi 27
Araneae Ptepi 32
Araneae Ptepi 29
Araneae Ncrue 20
Araneae Ncrue 42
Araneae Smimo 13 B
Araneae Smimo 32 B
Araneae Smimo 15 BSV
Opiliones Nsp 2
Scorpiones Tserr 3
Araneae Ptepi 28
Araneae Ptepi 69
Araneae Ptepi 33
Araneae Ptepi 2
Araneae Ptepi 3
Araneae Ptepi 48
Araneae Ptepi 65
Araneae Ptepi 63
Araneae Smimo 14
Araneae Smimo 11 B
Araneae Smimo 17 B
Araneae Smimo 30 B
Araneae Smimo 21
Araneae Ptepi 40
Araneae Ptepi 41
Araneae Ptepi 35
Araneae Lhesp
Araneae Ncrue 46
Scorpiones Tserr 1
Araneae Ageni 6
Araneae Ageni 20
Araneae Ageni 7
Araneae Ageni 11
Araneae Ageni 22
Araneae Ageni 16 B
Araneae Ageni 24 POH
Araneae Ageni 8 POH
Araneae Ageni 19
Araneae Smimo 19 B
Araneae Ptepi 54
Araneae Smimo 20 B
Araneae Ptepi 20
Araneae Ageni 17
Araneae Ageni 4
Araneae Ageni 14 POH
Scorpiones Tserr 14
Araneae Ageni 12 OH
Araneae Ageni 15
Araneae Smimo 18
Araneae Smimo 40
Araneae Smimo 10
Araneae Smimo 23
Araneae Smimo 12 BS
Araneae Smimo 7 B
Araneae Smimo 6 B
Araneae Ptepi 4
Araneae Ptepi 58
Araneae Ptepi 55
Araneae Ptepi 68
Araneae Ptepi 23
Araneae Ptepi 70
Araneae Ncrue 44
Araneae Ncrue 12
Araneae Ncrue 14
Araneae Smimo 9 B
Araneae Smimo 45
Araneae Smimo 31
Araneae Ptepi 21
Araneae Ptepi 22
Araneae Ptepi 42
Araneae Ptepi 43
Araneae Smimo 5 B
Araneae Ptepi 1
Araneae Ageni 1
Araneae Ageni 2
Araneae Smimo 22
Araneae Ncrue 41 DM
Araneae Ptepi 57
Xiphosura Lpoly 2
Opiliones Nsp 5
Scorpiones Tserr 5a M
Scorpiones Mmart 8
Scorpiones Mmart 6
Scorpiones Tserr 6
Scorpiones Tserr 4
Scorpiones Mmart 7
Scorpiones Tserr 7
Araneae Smimo 4
Araneae Smimo 8 B
Araneae Ncrue 11 DM
Araneae Ptepi 5
Araneae Ptepi 6
Araneae Ageni 23
Araneae Ageni 9 OH
Scorpiones Tserr 11
Araneae Linte 1
Araneae Linte 4
Araneae Linte 5
Araneae Linte 2
Araneae Linte 3
Araneae Smimo 28 B
Araneae Ptepi 36
Araneae Ncrue 22 DM
Araneae Ageni 5 O
Araneae Smimo 44 B
Scorpiones Mmart 4
Xiphosura Lpoly 1
Opiliones Nsp 3
Araneae Ptepi 7
Araneae Ptepi 8
Araneae Ptepi 10
Araneae Ptepi 11
Araneae Ncrue 45
Araneae Smimo 29
Araneae Ageni 3 POH
Araneae Smimo 24
Araneae Smimo 33
Araneae Smimo 35 B
Araneae Smimo 26
Araneae Smimo 25
Araneae Smimo 37
Araneae Smimo 27
Araneae Ptepi 12
Araneae Ptepi 13
Araneae Ptepi 16
Araneae Ptepi 14
Araneae Ptepi 15
Araneae Ptepi 39
Araneae Ptepi 17
Araneae Ptepi 18
Araneae Ptepi 38
Araneae Ptepi 9
Araneae Smimo 16 B
Araneae Ptepi 37
Araneae Ageni 10 OH
Parasitiformes Irici 2
Parasitiformes Irici 6
Parasitiformes Irici 3
Parasitiformes Iscap 3
Parasitiformes Amacu 1
Parasitiformes Amacu 2
Parasitiformes Irici 1
Parasitiformes Irici 7
Parasitiformes Iscap 1
Parasitiformes Amacu 3
Araneae Ncrue 38 DM
Araneae Ncrue 39 DM
Araneae Ncrue 13
Araneae Ncrue 37 DM
Araneae Ncrue 40
Araneae Ncrue 4
Opiliones Nsp 1
Acariformes Turti 3
Araneae Ncrue 29
Araneae Ageni 18
Acariformes Turti 1
Scorpiones Tserr 16
Araneae Ncrue 32 D
Araneae Smimo 38
Araneae Ncrue 31 DM
Scorpiones Tserr 9
Scorpiones Tserr 8
Scorpiones Tserr 15
Scorpiones Mmart 9
Araneae Ncrue 33 DM
Araneae Smimo 36
Araneae Ptepi 56
Araneae Smimo 34
Araneae Ptepi 62
Araneae Ncrue 34 DM
Scorpiones Tserr 12
Acariformes Turti 5
Acariformes Turti 6
Acariformes Turti 4
Araneae Smimo 41
Araneae Smimo 42
Parasitiformes Irici 4
Parasitiformes Iscap 2
Parasitiformes Irici 5
Araneae Smimo 2
Araneae Smimo 3
Araneae Smimo 1 B
Acariformes Turti 2
Opiliones Nsp 4
Araneae Ptepi 64
Araneae Ncrue 35 DM
Scorpiones Mmart 3
Araneae Ncrue 27
Scorpiones Mmart 1
Scorpiones Mmart 5
Araneae Smimo 30
Araneae Smimo 43
Opiliones Nsp 6
Araneae Ncrue 36 M
Acariformes Turti 7
Crustacea Aast
Crustacea Pcamt
Crustacea Lvann
20
